# A Review of Adsorbents for Heavy Metal Decontamination: Growing Approach to Wastewater Treatment

**DOI:** 10.3390/ma14164702

**Published:** 2021-08-20

**Authors:** Archana Gupta, Vishal Sharma, Kashma Sharma, Vijay Kumar, Sonal Choudhary, Priyanka Mankotia, Brajesh Kumar, Harshita Mishra, Amitava Moulick, Adam Ekielski, Pawan Kumar Mishra

**Affiliations:** 1 Department of Chemistry, MCM DAV College for Women, Sector 36, Chandigarh 160036, India; agupta271984@gmail.com; 2 Institute of Forensic Science and Criminology, Panjab University, Chandigarh 160014, India; sonalkarl6@gmail.com (S.C.); priyankamankotia786@gmail.com (P.M.); 3 Department of Chemistry, DAV College, Sector-10, Chandigarh 160011, India; shama2788@gmail.com; 4 Department of Physics, National Institute of Technology Srinagar, Srinagar 190006, India; vj.physics@gmail.com; 5 Post Graduate Department of Chemistry, TATA College, Jharkhand, Chaibasa 833202, India; bkumar@espe.edu.ec; 6 Centro de Nanociencia y Nanotecnologia, Universidad de las Fuerzas Armadas ESPE, Av. Gral. Rumiñahui s/n, Sangolqui 171103, Ecuador; 7 Smart Society Research Team, Faculty of Business and Economics, Mendel University in Brno, 61300 Brno, Czech Republic; harshitamishra1088@gmail.com (H.M.); amitavamoulick@gmail.com (A.M.); 8 Department of Production Engineering, Warsaw University of Life Sciences, 02-787 Warsaw, Poland; adam_ekielski@sggw.edu.pl; 9 Faculty of Business and Economics, Mendel University in Brno, 61300 Brno, Czech Republic

**Keywords:** heavy metal, adsorption, polymeric adsorbents, bioadsorbent, wastewater

## Abstract

Heavy metal is released from many industries into water. Before the industrial wastewater is discharged, the contamination level should be reduced to meet the recommended level as prescribed by the local laws of a country. They may be poisonous or cancerous in origin. Their presence does not only damage people, but also animals and vegetation because of their mobility, toxicity, and non-biodegradability into aquatic ecosystems. The review comprehensively discusses the progress made by various adsorbents such as natural materials, synthetic, agricultural, biopolymers, and commercial for extraction of the metal ions such as Ni^2+^, Cu^2+^, Pb^2+^, Cd^2+^, As^2+^ and Zn^2+^ along with their adsorption mechanisms. The adsorption isotherm indicates the relation between the amount adsorbed by the adsorbent and the concentration. The Freundlich isotherm explains the effective physical adsorption of the solute particle from the solution on the adsorbent and Langmuir isotherm gives an idea about the effect of various factors on the adsorption process. The adsorption kinetics data provide valuable insights into the reaction pathways, the mechanism of the sorption reaction, and solute uptake. The pseudo-first-order and pseudo-second-order models were applied to describe the sorption kinetics. The presented information can be used for the development of bio-based water treatment strategies.

## 1. Introduction

**Figure 1:**
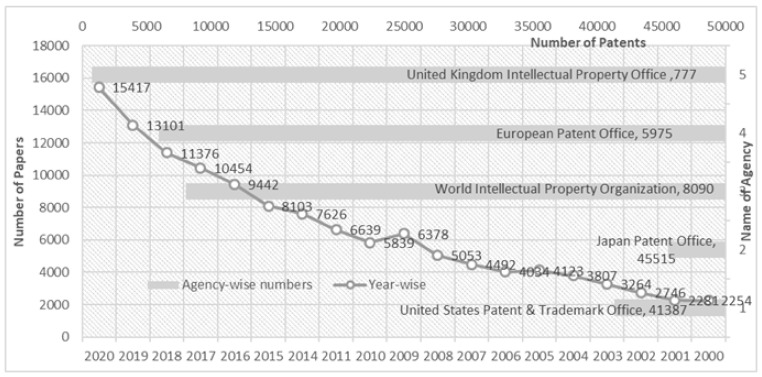
Number of research papers and patents available on the use of various materials for wastewater treatment per year from 2000 to 2020 from Scopus with the keywords “wastewater treatment”.

Many pollutants are entering water bodies due to the continuous increase in the number of global inhabitants, manufacturing units, unplanned urbanisation, cultivation ventures, and the use of chemicals [1]. In today’s scenario, researchers are working on the effective removal of various pollutants such as heavy metals, synthetic colour, sediments, chemicals, radioactive, pharmaceuticals, and other waste material from the natural cycles [2]. Among all the contaminants, heavy metals are the most prevalent contaminant found in water. Heavy metals are described as elements with a particular density greater than 5 g/cm^3^ [3]. This classification covers critical elements for trace concentrations (for example iron, vanadium, cobalt and copper, manganese, zinc, strontium, and molybdenum). If the threshold amount is surpassed, however, multiple damage in living systems may be found [4]. Heavy metals are found in the atmosphere from both natural (e.g., soil erosion, weathering of the earth’s crust, volcanic eruptions) and anthropogenic sources (e.g., mining and mineral beneficiation, industrial effluents from cement, food, fiber, paper, electronic operations, chemical formulations for plague control, and so on) sources [5]. Since heavy metals do not decay in the atmosphere, their accumulation in environmental compartments (such as air, soil, and waters) could contribute to their migration into food and water intended for human consumption in the long run [6]. This is a significant environmental issue, prompting scientists to focus on new technologies that will enable heavy metals to be removed from polluted environmental supplies. Heavy metals act as a poison to metabolic activities and enzyme inhibitors [7]. These toxic metals are not degraded biologically and tend to accumulate in living beings, resulting in different illnesses and disorders. Heavy metals leak into nature by various modes like combustion of coal, sewage waste-water, and emission of toxic gases by automobiles, battery manufacturing industries, activities related to extraction of minerals, leather industries, alloy industries, and the consumption of non-renewable energy resources [8]. Various methods like filtration, precipitation through chemical methods, neutralisation, ion exchange, and adsorption are used to bind heavy metal ions from the wastewater discharged from many industries [9,10,11,12]. To date, a large number of published papers on wastewater treatment have increased progressively since 2000 (Figure 1).

The removal of heavy metal ions has always been a challenge for scientists as effective adsorbents are required for their elimination. Adsorbents have many benefits over traditional chemical sorbents in water treatment systems: biodegradability in natural/environmental settings, high abundance in nature, high surface-area, a greater tendency to adsorb such metal ions, appropriate pore dimension and volume, more mechanical strength, compatibility, they are easily available, they are easily renewable, they have a cheap cost, their eco-friendliness, they have easy manufacturing methods, and they are more specific in nature [13]. These structures have a high surface area to volume ratio and multiple active binding sites on their surface, enabling heavy metals to be maintained effectively under some conditions (e.g., –COOH, –NH_2_, –OH, –SH groups) [14]. Adsorption is generally dependent on the association of hydroxyl, amino, and carboxylic groups on the adsorbent’s surface with metal ions such as Cu^2+^, Pb^2+^, and Cr^3+^ cations [15,16].

In the past two decades, the adsorption of heavy metals with environment-friendly and economical materials like agricultural, industrial, or urban residues has come up with a promising technology in the removal of pollutants from aqueous discharge [17]. Hence, a lot of research has been reported by many authors using economical adsorbents such as lignin, bark, chitosan, clay, zeolite, activated carbons, synthetic polymeric adsorbents, and so on [18]. The present review focuses on the use of various types of adsorbent for the removal of heavy metal ions. There are not many research papers that use multiple types of adsorbents in a single frame to eliminate them. The classification of various types of adsorbent like natural, synthetic and modified adsorbents have been covered in this review which otherwise was not included in any of the reviews published before [19,20,21,22,23]. 

Adsorption is a commonly proposed technique since it is highly effective in the extraction process and is easy to apply; adsorbents are available in different ranges and are cheaper in cost [24]. Several chelating resins were synthesised by the polymerisation of traditional chelating monomers i.e., methacrylic acid, acrylamide, vinylpyridine and vinylimidazole [25,26]. Researchers synthesised a polymer by reacting this with ligands having a low molecular weight which resulted in a new polymer with some functionality or new group in the modified polymer [27,28]. Additionally, the functionalisation of the polymer matrix results in the formation of new chelating resin [29]. Because of the existence of reactive hydroxyl and amino moieties, chitosan is a natural biopolymer that is abundant in nature and has the ability to adsorb a considerable amount of metals. Furthermore, modification of chitosan through physical and chemical techniques were able to increase its sorption abilities for metal ions like As^3+^ or As^5+^ [30,31,32], Cr^6+^ [33,34,35], Pb^2+^ [36,37], Cu^2+^ [38,39,40], Hg^2+^ [41,42,43], Cd^2+^ [44,45,46], Ni^2+^ [47,48,49], Zn^2+^ [50,51], Ag^+^ [52,53], Co^2+^ [54], Mn^2+^ [55], U^6+^ [56,57], V (3+,4+ and 5+) [58], and Pt^4+^ [59]. Similarly, Jiang et al., synthesised polystyrene-supported chitosan beads cross-linked with glutaraldehyde for rapid extraction of copper ions [38]. Yan et al. (2010) studied whether the coagulation procedure was useful in the removal of arsenic (As), and found that Al and Fe salts were effective coagulants for extraction. Furthermore, since 2000, the amount of data available on adsorption in water treatment has steadily risen (Figure 1), suggesting a rise in research interest in this area [60].

## 2. Heavy Metals

These refer to the elements with an atomic weight higher than water. Even at low concentrations, these are lethal. The heavy metals present in water are the main ecological concern due to their accumulation and non-degradability on their own [61,62]. Various sources like paper manufacturing units, fungicide, insecticide, lather industry, mining operations, and electroplating industries release heavy metals into the water resources which are highly toxic and cannot be destroyed on their own [63]. Heavy metal ions are even more toxic when present in hydrated form. Therefore, their toxicity increases in waterbodies affecting the life cycle of many living organisms. Therefore, the extraction of toxic metals is essential to eliminate their hazardous effect on aquatic life. The World Health Organisation (WHO) and Environmental Protection Agency (EPA) have laid down certain permissible limits for the discharge of heavy metals into nature to reduce water pollution. There are mainly four heavy metals namely lead (Pb), cadmium (Cd), mercury (Hg), and inorganic arsenic (As) which concern individual health. The inability to metabolise metals in the body is one of the most fundamental concerns among problems associated with heavy metals. Heavy metal ions have a detrimental effect on human health and the environment. These effects include reduced growth of organs, cancer, disruption of the body’s nervous system, disruption of the body’s defense system, and in severe cases include life-threatening. Heavy metals can precipitate and accumulate in the bone, fat, muscle, and other tissues which can lead to many diseases and complications in the body [64]. Heavy metals are mostly used in different industries like paper, tanneries, agriculture, and so on and their broad applications result in national economic growth and the development of civilisation. The excessive presence of metal ions in the body beyond the admissible value damages the biological process of cells [65]. These toxic metals become the reason for the decrease in the functioning of the cerebrum and nervous system, enhance blood pressure, decrease the amount of blood in the body, affect lungs, kidneys, and other parts of the body, and cause frailty and a lapse of memory as well as an increase of allergies [66]. Some of the heavy metals and their bad effects are summarised below.

### 2.1. Cadmium and Chromium

Cadmium and chromium are present in the form of natural deposits in industrial waste. It is the main component of industries like plating, battery industry used for making cadmium-nickel batteries, fertilisers made of phosphate, stabilisers, and alloys. While chromium is used in industries related to lather, tanneries, electroplating, fabric industry, and so on [67,68]. Both elements are extremely dangerous and accumulate in the environment. Cadmium causes chronic kidney failure, cancer in human beings, softening and weakening of bones. Its symptoms are deficiency of vitamin D, itai–itai disease, diseases related to respiration, diseases related to gastrointestinal loss of red blood cells which hinder the function of calcium in the body of living beings. Chromium causes genotoxicity, skin inflammation, liver and kidney damage, and alopecia. As both metals have dangerous effects, it is necessary to remove both metals before they enter water bodies [69,70].

### 2.2. Nickel and Lead

Nickel is a metal, silver in color, and cannot be destroyed naturally. The most important sources of nickel heavy metal are printing, electroplating industries, silver and gold plants, battery manufacturing units, and alloy units. The side effects of nickel involve problems like dry cough, chest ache, sickness, diarrhea, skin eruption, pulmonary fibrosis, gastrointestinal ache, and renal edema, etc. [71,72,73]. Lead is a soft metal that occurs in the form of sulphide, cerussite (PbCO_3_), and galena (PbS). The most important causes of lead waste are lead-acid batteries, electroplating industries, electrical manufacturing units, steel production units, explosive manufacturers. The major side effects of lead waste are kidney and nervous system damage, mental abnormality, and cancer in the human body [74,75,76].

### 2.3. Copper and Zinc

Copper and zinc are poisonous metals if present in high concentrations in nature. Both the elements are required for the synthesis of enzymes, bone and tissue growth [77,78]. Zinc helps in controlling the chemical reactions in living beings and physiological processes [79]. The main sources of copper and zinc metals are electroplating industries, paper and pulp industries, steel making, metallurgy, chemical industries, printing circuit board, electroplating industries, paints, fertilisers, etc. [80,81]. Zinc creates health problems like headache, nausea, skin irritation, fever, anaemia, while copper causes hair loss, anaemia, kidney damage, and nuisance [82].

## 3. Adsorption Mechanism

**Figure 2:**
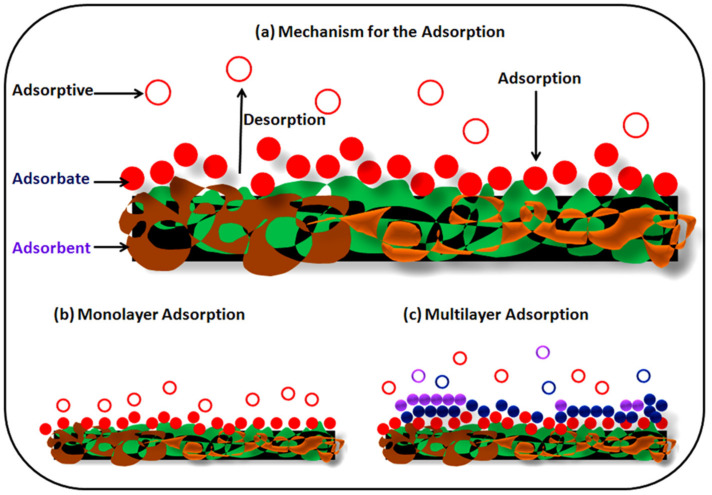
(**a**) General mechanism for the adsorption, (**b**) monolayer adsorption, and (**c**) multilayer adsorption.

The adsorption process is the most efficient and admirable process for the treatment of toxic metals present in wastewater. In this process, toxic material is shifted by physical or chemical means onto the available surface of the adsorbent (Figure 2) [83]. The adsorption process is a cheap method and has a very low operating cost, and causes less contamination during the extraction process of toxic metal in comparison to conventional methods. In the adsorption methods, sorbents can be regenerated as well as reused several times for effective removal, and hence are considered to be an environmentally friendly method [84]. The major characteristics required for the choice of adsorbents are price effectiveness, large surface area, pore size distribution, presence of functional moiety, and polar characteristics of the sorbent which determines the efficiency of adsorption methods [85]. It is, therefore essential to understand the adsorption process. Adsorption is a mass transport method of solute present in the solution and accumulated onto the surface of the adsorbent which is generally a solid substance [86]. There are two kinds of force i.e., physical and chemical interactions that exist between adsorbent and adsorbate. Physical forces are weak and adsorbed molecules can be attached to adsorbents anywhere, meaning physical forces are non-specific in nature. Chemical adsorption is specific in nature and adsorbate binds to adsorbents through the covalent or electrostatic bonds. In the case of physical adsorption, forces are Van der Waals, dispersion interactions, and hydrogen bonding.

## 4. Adsorption Modeling

### 4.1. Adsorption Kinetics

Adsorption kinetics data are expected to create an efficient and reliable design model for the removal of pollutants from aqueous media. Kinetic models explain the sorption process and method used. It also explains the speed with which adsorption sites of adsorbent are occupied and the number of unoccupied sites. The adsorption kinetic is commonly used to predict the various adsorbent’s sorption ability. Kinetic modelling actually gives information about adsorption mechanisms and possible rate-controlling steps such as mass transport or chemical reaction processes. Kinetic models help us to find out the metal extraction rate which finds the time to attain the equilibrium. The rate of the removal of solute depends upon the physical or chemical properties of the sorbent and also on the various parameters that affect the rate of adsorption. Usually, the rate of sorption of toxic ions becomes better with time until the equilibrium was achieved between the amounts of adsorbates in the solid phase and the amounts of adsorbates present in the liquid. Mostly, the rate of adsorption is fast in the start and steadily slows down as equilibrium is reached. The time to obtain equilibrium varies with the adsorbates, adsorbent, initial concentration, and the various parameters that affect the solution. There are different kinds of model used by researchers to study the sorption process such as Lagergren’s pseudo-first-order (PFO), pseudo-second-order, Elovich kinetic equation, and parabolic diffusion model. Among the entire models, the rate of sorption was generally studied through the Lagergren pseudo-first-order model. At present, the pseudo-second-order (PSO) model has been generally used for the sorption process because of its excellent illustration of the practical facts for all sorbent sorbate systems. For this, PFO and PSO kinetic models will be utilised to describe the adsorption kinetics of heavy metal ions onto adsorbent, which was defined as follows.

#### 4.1.1. Lagergren’s Pseudo-First-Order Kinetics

The pseudo-first-order kinetic model has been mostly used to speculate the rate of sorption of metal which is described as:

dq/dt = k_1_ (q_e_ − q) (1)

The term q is the quantity of metal sorbed in a particular time (mg/g), q_e_ is the quantity of metal sorbed at equilibrium time (mg/g), k_1_ is pseudo-first-order rate constant (g mg^−1^ min^−1^). We integrate Equation (1) by using boundary conditions q = 0 at t = 0 and q = q at t = t, then solve Equation (1) as below:

In (q_e_/q_e_ − q) = K_1_ T (2)

Thus, the rate constant k_1_(min^−1^) can be obtained from the graph of In(q_e/_q_e_ − q) versus time [87].

#### 4.1.2. Pseudo-Second-Order Kinetics

The sorption kinetics can also be studied by the pseudo-second-order model. PSO model assumes that the rate of adsorption of solute is proportional to the available sites on the adsorbent. Equation (3) shows the linear form of PSO.

dq_t_/dt = k_2_ (q_e_ − q_t_)^2^ (3)

where K_2_ (g mg^−1^ min^−1^) is the is the equilibrium rate constant of pseudo second-order adsorption. On integrating (3) and noting that, q = 0 at t = 0, the following equation is obtained.

t/q_t_ = 1/k_2_ qe^2^ + 1/q_e_ t (4)

The equilibrium adsorption capacity, is obtained from the slope and is obtained from the intercept of linear plot of t/versus t.

## 5. Adsorption Isotherm

The adsorption isotherm model is a valuable method for determining the theoretical optimal adsorption power as well as the potential interactions between adsorbents and adsorbate [88]. Sorption isotherms are mathematical models that illustrate the allocation of metals in between adsorbate and adsorbent. The distribution of metals in between adsorbate and adsorbent depends upon the nature of the adsorbent whether it is homogeneous or heterogeneous, the type of exposure, and the bonding between adsorbent and adsorbate. In this study, models of adsorption isotherm of adsorbents were studied by utilising Langmuir and Freundlich. The Langmuir isotherm assumes that all binding sites for the adsorbate have an equal affinity, resulting in the creation of a monolayer of adsorbed molecules [89]. The Freundlich isotherm, on the other hand, primarily defines adsorption into heterogeneous surfaces with adsorption sites of different affinities which were defined as follows [90]. Table 1 shows the linear and non-linear expressions of the Langmuir and Freundlich isotherms.

### 5.1. Freundlich Isotherm

The Freundlich equation is used to present sorption value [91]. The Freundlich isotherm explains the physical sorption of the solute particle from metal solution to the adsorbent. The Freundlich adsorption isotherm is explained as below:

q_e_ = K_f_ C_e_^1/n^ (5)

The term q_e_ is the quantity of solute in milligrams sorbed by one gram of sorbent at equilibrium (mg/g), C_e_ is the equilibrium amount of adsorbate in solution after sorption (mg/L) and K_f_ is the Freundlich constant (L/mg): n is the heterogeneity factor. Adsorption data well fit to Freundlich isotherm indicates that solute sorbed on the exterior of the sorbent is forming many layers. The suitability of the Freundlich equation is examined by logq_e_ against log*C*_e_ by the use of the logarithmic form of Equation (5).

log q_e_ = log K_f_ + 1/n log C_e_ (6)

The above equation is a straight-line having intercept log K_f_ and slope equal to 1/n.

### 5.2. Langmuir Isotherm

The Langmuir adsorption isotherm was explained on the basis of subsequent presumptions

- Adsorbent surface has fixed vacancy on the adsorbent surface with the same energy.
- Only monolayer sorption occurs on the surface of sorbent which is reversible.
- Available empty sites having the same size and shape on the adsorbent surface [91].

q_e_ = q_0_ K_L_ C_e_/(1 + K_L_ C_e)_ (7)

The term q_e_ is the quantity of solute in milligrams sorbed by 1 g of sorbent at equilibrium (mg/g), C_e_ is an equilibrium amount of adsorbate in solution after sorption (mg/L), q_0_ represents highest sorption value (mg/g), and also known as the experimental Langmuir constant and K_L_ is the experimental Langmuir constant (L/mg) The q_0_ represents the presence of the highest number of vacancies on the sorbent. The constant K_L_ represents the Langmuir constant indicates the same adsorbate attraction for all surface sites.

Arrange the Equation (7) we obtain:

C_e_/q_e_ = 1/q_0_K_L_ + C_e_ q_0_(4) (8)

the graph between C_e_/q_e_ vs. C_e_ gives a straight-line having slope 1/q_0_ and intercept 1/q_0_K_L_.

### 5.3. Adsorption Thermodynamics

For metal ion sorption, the temperature is important as far as thermodynamic adsorption is concerned. Usually, endothermal and exothermic sorption processes are the two general types that exist. These are determined by the increase or decrease of the temperature throughout the adsorption process. Thermodynamic functions such as entropy, enthalpy and free changes in energy can be determined using the Van’t Hoff equation during the adsorption process as given below:

d(lnKeq)/dT = ΔH/RT^2^ (9)

where Keq = qe/C. Keq is equilibrium constant. The change in free energy could be evaluated with the help of Equation (10):

ΔG^0^ = ΔH^0^ − TΔS^0^ (10)

Gibbs free energy change is related to equilibrium constant (Equation (12)):

ΔG^0^ = −RTlnKeq (11)

By combining Equations (10) and (11), Equation (12) is obtained:

ln Keq = −ΔH^0^/RT + ΔS^0^/R (12)

The change in enthalpy and entropy of the adsorption process could be evaluated from the slope and intercept of a line obtained by plotting ln Keq vs. 1/T. 

If H^0^ is positive, the reaction is endothermic, which means that as the temperature rises, so does the adsorption efficiency. If S^0^ is positive, however, the randomness at the moment of adsorption rises, and if G^0^ is negative, adsorption is spontaneous or favorable [92]. There are two types of adsorption process: spontaneous and non-spontaneous.

Spontaneous reactions: ΔH^0^ < 0 ΔS^0^ > 0 ΔG^0^ < 0

Non-spontaneous reactions: ΔH^0^ > 0 ΔS^0^ < 0 ΔG^0^ > 0

Gibb’s free energy, enthalpy, and entropy changes are important design factors for assessing the performance and predicting the mechanism of an adsorption separation process, as well as one of the fundamental needs for characterisation and optimisation Table 1 show typical values for thermodynamic parameters for adsorption of heavy metal ions on adsorbents [93].

## 6. Factors Affecting the Adsorption Process

### 6.1. Effect of pH on Adsorption

The pH of the mixture plays a significant role in sorption. It affects the metal ion’s adsorption on the adsorbent. The pH effect of the solution may be demonstrated by altering the bio sorbents surface loads and the ionising degree in an aqueous solution of the metal ions. Therefore, it is essential to evaluate the effect of pH on adsorption. Furthermore, the presence of a significant number of H^+^ and H_3_O^+^ ions in water competes with metal ions for adsorption. At a moderate pH, the rate of adsorption rises because electrostatic repulsions are reduced due to deprotonation of the active sites [94]. Several other investigations have found that adsorption of heavy metal ions is better at moderate pH levels than at lower pH levels. For example, adsorption of Pb (II) and Cu (II) on the surface of chitosan/TiO_2_ nano-fibers was maximum at pH 6.0 and lowest at pH 2.0 to 4.0 [95]. A comparable study found that increasing the pH from 4.0 to 6.0 increased the percentage of Cd (II) adsorption, which then remained constant (97%) at pH 9.0. After that, lowering the pH to 11.0 lowered the adsorption proportion to 80% [96]. According to the preceding explanation, a moderate pH is suitable for heavy metal ion removal because the sorbent surface is deprotonated at this pH, resulting in an increase in negatively charged sites. This results in higher electrostatic attractions between the adsorbent surface and metal cations, which increases adsorption capacity. However, at lower pH, there is an increase in positively charged adsorption sites, which leads to an increase in repulsive interactions between the adsorbent surface and positively charged metal ions, lowering metal ion adsorption [97].

The sorption of Pb^2+^ and Hg^+^ ions by the modified polyglycidyl methacrylates were investigated under a non-competitive environment at various pH values and times [98]. It was found that Hg^2+^ and Pb^2+^ ions extraction by the resins increases as the solution pH values are increased, particularly in the pH range of 1–5.7 for Hg and for lead 1–4.8 higher pH promotes electrostatic attraction between positively charged metal ions and negatively charged biosorbent surfaces, increasing adsorption efficiency. This slow adsorption may be explained by the presence of electrostatic repulsion between these positively charged species on the adsorption surface and metal ions [99].

### 6.2. Effect of Contact Time

It is found that the rate of adsorption increased with an increase in the time of reaction until the equilibrium is obtained between the number of absorbents sorbed on the absorbents and the number of sorbates that remain in the solution. In general, adsorption occurred quickly in the preliminary phases and steadily slows down as the equilibrium state occurred between metal in the liquid and solid phase. Metal ions having different concentrations have achieved the equilibrium at a different time and this depends upon the concentration of metal ions, adsorbents, initial concentration, and the temperature of the solution. Gupta et al., studied the removal of lead ions by three different amine adsorbents, PAN-EDA, PANTETA, and PAN-TEPA. It was found that removal efficiency gets increased with time. It was found that at low pH, the extraction of these metal ions decreased due to the competition between metal and H^+^ ions in solution towards the adsorbent during the removal process. Also at higher pH i.e., in basic conditions precipitations of metal ions as hydroxides species such as soluble Hg(OH)^+^ or insoluble precipitate, Hg(OH)_2_ formation took place.

### 6.3. Effect of Other Co-Existing Ions

The investigation of the effect of coexisting ions is most important in adsorption studies as industrial wastewater consists of different anions and cations. There may be rivalry among the different ions for the same adsorption sites. It is preferable to prepare a multifunctional sorbent capable of extracting different forms of metal ions. Kesenci et al. [100] synthesised poly(ethylene glycol dimethacrylate-acrylamide) copolymer for extraction of (lead mercury and cadmium) metal ions in an aqueous solution and the order of metal uptake was found to be lead > cadmium > mercury. For a 1.0 mg/L concentration of each metal, it was discovered that cadmium adsorption was higher in the mixture (0.52 mmol) than in the individual solution (0.37 mmol) [100].

### 6.4. Effect of Sorbent Dose

The sorbent amount is another significant feature in the determination of the removal efficiency of the sorbent. With an increase in sorbent amount, the percentage of metal extraction increases. This may be due to the presence of sorption space, to which adsorbate will bind. The determination of adsorbent dose provides an idea regarding the least amount of sorbent requires for the sorption process. Kumari et al. [101] reported that with an increase in the dose of chitosan the removal of chromium (VI) is increased. The removal efficiencies were studied by taking the different amounts of adsorbent in the range from 0.1 to 0.4 g. The maximum removal would take place with the 0.4 g of adsorbent [101]. The removal efficiencies were investigated using various amounts of adsorbent ranging from 0.1 to 0.4 g. For 0.4 g of adsorbent, the optimal removal will be achieved [101].

### 6.5. Regeneration of Adsorbent

**Figure 3:**
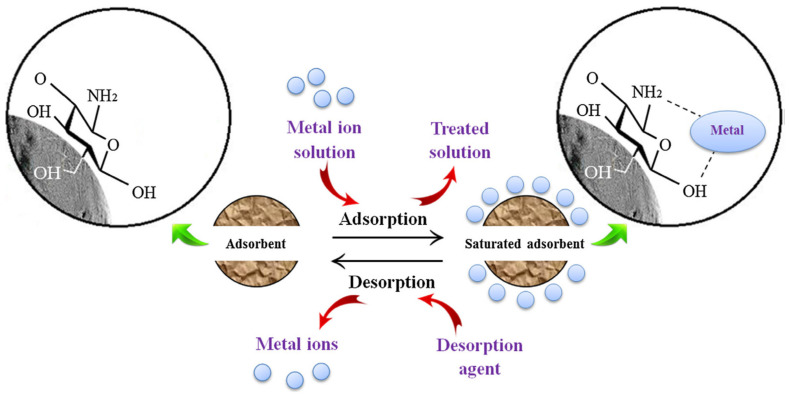
Schematic representation of regeneration procedure of chitosan adsorbent. Reproduced with permission from [103].

The regeneration of adsorbents will indicate the reuse of adsorbents and will increase the effectiveness of an adsorbent. Regeneration makes the reaction an economic reaction. Regeneration could be done by using solvents such as EDTA, HCl, HNO_3_, NaCl solution and NaOH solution are reported in research papers for regeneration studies [102]. Regeneration of chitosan-based adsorbents employed in heavy metal adsorption was reviewed in [103]. The regeneration of a chitosan-based adsorbent for the adsorption of heavy metal ions from the wastewater is shown schematically in Figure 3. The regeneration performance of clay-based adsorbents for the removal of industrial dyes was also reviewed in [104].

Qu et al. [105] suggested that chelating polymer might be recovered by using 2% thiourea in 0.1 mol/L HCl and the resin could be reused with the same efficiency. Similarly, Saglam et al. [106] suggested that PHEMA microbeads containing the thiazolidine group can be recovered by treating them with HCl (0.05 M), implying that the resin could be used three or four times in adsorption–desorption cycles without losing its sorption capacity. Different techniques like scanning electron microscopy (SEM), energy-dispersive X-ray spectroscopy (EDX), X-ray diffraction (XRD) analysis, electron paramagnetic resonance (ESR), X-ray photoelectron spectroscopy, and atomic absorption spectroscopy are being used to provide information about the adsorbent [106].

### 6.6. Types of Adsorbent

Adsorption has certain advantages over conventional methods such as minimising chemical and biological sludge, low cost, high efficiency, regeneration of adsorbents, and the possibility of metal recovery. A large number of natural adsorbents were synthesised for the extraction of heavy metal ions. Adsorption efficiencies of natural adsorbents are mentioned in Table 2.

#### 6.6.1. Bark and Other Resources with Ample Tannin Content

Agricultural origins contain tannin, lignin, cellulose, hemicellulose, extracts, lipids, proteins, sugars, water, and a variety of other compounds with different functional groups [114,115]. The bark is obtained as a by-product of the wood manufacturing units. In bark, tannin is present in a large amount and this organic substance is very efficient for the extraction of heavy metal ions. Tannin contains polyhydroxy polyphenol moieties which help in the adsorption of metal ions [18]. Masri et al. [116] were the first to report the removal of cadmium, mercury, and lead using black oak and Douglas fir bark respectively. Cadmium and chromium were removed from wastewater employing coffee, waste tea, and walnut shell [117]. Randall et al. [118] also investigated the elimination of heavy metal ions like cadmium, lead, copper, and mercury by using bark, formaldehyde polymerised bark, and peanut skins. Adsorption capacities for bark have been reported to be in the range of 1.5–27.6 mg/g for various tannin-containing materials. However, the problem of discoloration of water arose due to the presence of soluble phenols. Chemical pre-treatment of bark with acid, base, or acidified formaldehyde has been shown to overcome the problem of discoloration in water without appreciably affecting ion removal capacity. Another by-product that is obtained from the wood industry is sawdust [119]. Adsorptions of Cu, Cr, Ni, Cd, and Pb have been reported by investigators using oak, red fir, and cedar sawdust in the capacities 0.0683–0.0982 meq/g [120]. Thus, the bark is helpful in the elimination of toxic ions from water bodies and is the main possible option due to economics along with its large accessibility. In another study, S. Kumari et al. [101] used Andean Sacha inchi shell biomass (SISB) to expel Pb^2+^ and Cu^2+^ from wastewater. The better suit isotherms for both Pb^2+^ and Cu^2+^ were Langmuir > Freundlich, with maximal adsorption capacities of 17.066 and 9.699 mg/g at 323 K, respectively [101].

#### 6.6.2. Lignin

**Figure 4:**
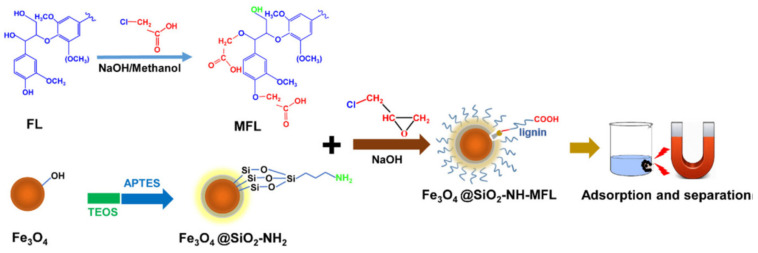
A schematic process flow diagram illustrating the synthesis of the lignin-based hybrid magnetic nanoparticles. Adapted with permission from Reference [125].

Lignin is derived from black liquor and used as a devastating ingredient in paper processing units. Srivastava et al. investigated the adsorption of zinc and lead on the surface of lignin [107,121]. It is a very cheap adsorbent as compared to activated carbon for the elimination of toxic ions. The factor which was responsible for the sorption competence of lignin is polyhydric phenols and additional functionality present on the surface. The ion exchange method was used to remove heavy metal ions by the use of lignin. In a different experiment, Masri and Friedman produced sulphuric acid-functionalised lignin that can adsorb mercury at concentrations of up to 150 mg/g [116]. Celik and Demirba synthesised a natural sorbent from improved lignin material for the extraction of lead, cadmium, copper, and zinc metal solutions prepared in a lab with an initial concentration of 50 mg/L. To prepare this adsorbent, black liquor specimens were collected from the pulp industrial unit of the Mediterranean Foundation of SEKA in Turkey. The maximum adsorption value was found to be 11.3 mg/g for Zn (II), 17.5 mg/g for Pb (II), 7.7 mg/g for Cd (II) by the lignin. The time period to attain the highest adsorption was 4 h for lead ions with 96.7% adsorption capacity and for Zn (II) ions 10 h with 95.0% sorption efficiency. It was found that adsorption values improved with pH indicating that metal ions undergo ion exchange with lignin [122,123]. Carboxymethyl lignin nanoparticles were prepared through a microwave heating method and antisolvent two-stage processes help to modify the extraction ability of carboxymethyl functionalised lignin and decrease the solubility of the resultant adsorbent. The adsorbent was used to extract lead ions and the maximum sorption value was found to be 333.26 mg/g compared to traditional adsorbents prepared from lignin. The effect of pH value was also studied in the range 2.04–7.06 for carboxymethyl lignin nanoparticles (CLNPs) as well as lignin nanoparticles (LNPs). It was found that with an increase in pH up to 6.03, CLNPs showed much higher adsorption compared to LNPs [124]. At lower pH values, the rate of sorption decrease more than LNPs due to the presence of a large number of H+ ions in the solution which combines with the -COO- group to form –COOH. Also, at pH value 6.03, carboxymethyl lignin nanoparticles showed better adsorption phenomena (217.21 mg/g) than lignin nanoparticles (51.61 mg/g). The regeneration ability of carboxymethyl lignin nanoparticles was better than lignin nanoparticles. Lignin/titanium oxide nanoparticles were synthesised for the extraction of metal ions as well as to study the photocatalytic features of the adsorbent [124]. In this paper, four types of lignin/nanomaterials such as corn stalks and cotton stalks, wood chips, and poplar wood chips nanotubes represented as L-TNTs CNL-TNTs, WL-TNTs, and PL-TNTs were used to extract lead, copper, and cadmium ions from the garbage. From the adsorption isotherm of materials, it was found that the extraction ability of WL-TNTs was more for lead, copper, cadmium compared to PL-TNTs. The highest sorption values were 677.6 mg/g 258.2 mg/g and 308.5 mg/g for Pb (II), Cu (II), and Cd (II), respectively by the WL-TNTs. PL-TNTs had shown 546.8 mg/g, 169.3 mg/g, and 245.4 mg/g for Pb (II), Cu (II), and Cd (II) extraction. An adsorption kinetic study showed that WL-TNTs rapidly removed metal ions Pb (II), Cu (II), and Cd (II) 87%, 71%, and 73%, respectively, within 5 min. After 120 min, there was no increase in metal ions adsorption. Zhang et al. prepared a novel environmentally friendly and cheaper adsorbent based on a magnetic core and mesoporous silica shell conjugated with the modified lignin (Figure 4) [125]. Lignin based-adsorbents were reviewed recently by Supanchaiyamat and co-workers, with the focus on lignin, its modification, and carbon materials derived from this abundant feedstock [126].

#### 6.6.3. Chitosan and Seafood Processing Wastes

Chitin is also present in large quantities in nature and its origin is from the body of some invertebrate animals like crabs, different arthropods, and from various fungi. Chitosan is a deacetylated type of chitin and broadly utilised as an adsorbent particularly for wastewater treatment [127]. Yang and Zall reported that chitosan metal uptake was more than chitin due to the free amino moieties present during deacetylation [128]. Chitosan was found to have extraordinary holding efficiency at 1 mmol metal/g for the majority of metals in comparison to bark-activated sludge, poly(p-amino styrene), and other sorbents [116]. Kurita et al. noticed that the sorption capacity of chitosan changes with certain parameters like crystalline nature, hydrophobic nature, percentage of deacetylation, and free amino moiety [129]. Polysaccharides, after 50% deacetylation still have high solvency in water and present trouble for practical use. Therefore, in order to ensure the high stability of deacylated polysaccharides, they are crosslinked with glutaraldehyde, but it may cause a decrease in the sorption capacity of chitosan [130]. The effectiveness of crosslinked chitosan beads was also discussed by Rorrer et al., (1993). The adsorbent obtained in the form of beads had more surface area than chitosan powder. Adsorption capacities of cadmium on chitosan powder and beads were found to be more significant capacity, a faster rate of adsorption, and enhanced ease of operation over different structures. Instead of being dissolved in an acid medium, chitosan is also non-porous in nature [108]. Bertoni et al. used chitosan polysaccharides for the extraction of molybdate ions from polluted water [131]. Chitosan was a cationic polysaccharide and its hydroxyl group was used to bind the Mo (VI) metal anions. Chitosan has a high affinity to bind Mo(VI) at pH 2.7. The adsorption capacity reached 100% after two cycles and adsorption time was found to be 160 min and the volume of treated water during this time period was 1.44l in the first cycle. The adsorption capacity of these polysaccharides was found to be 265 mg/g at 20 °C at an of pH 2.7. Hsien et al. recommended N-acylation as a method for intensifying permeability within chitosan [132]. Despite the fact that chitosan was excellently adsorbed by the ions in its original state, its adsorption capacity could be enhanced by reacting with different functional groups, for example, organic acids, onto the chitosan backbone [133]. Some functional groups grafted to chitosan were amino acid; esters, pyridyl, substituted pyridine rings, oxo 2-glutaric acid, and polyethyleneimine enhance the adsorption capacity. Chitosan derived from shrimp shells was used to remove Cu^2+^ (98.97%), Cr^4+^ (37.51%), and Fe^2+^ (65.2%) from industrial water. After that, the mixture was rotated with a speed of 7000 revolutions per minute for 5 min. Cu^2+^ (98.97%) had a higher affinity than Fe^2+^ (65.2%), Cr^4+^ (37.51%), and Zn^2+^ (37.51%) among the four metal ions studied (86.15%) [134].

**Figure 5:**
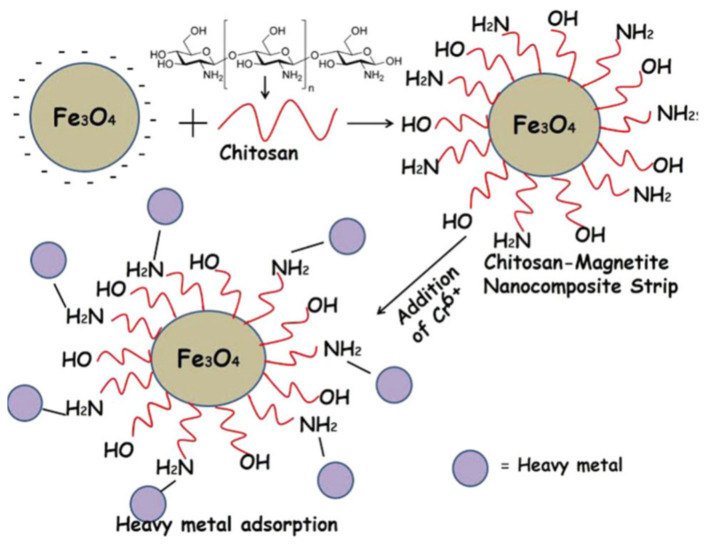
Schematic representation of removal mechanism of chromium ions by chitosan-magnetite nanocomposite strip. Reproduced with permission from [137].

In addition, the researchers employed 0.1 g of the aforementioned adsorbent for chromium metal ions removal and obtained 19.28 mg/g removal efficiency by using shrimp chitosan. The removal efficiency was essentially boosted by increasing the adsorbent dosage and centrifuge speed [101]. Because of the presence of different functional groups, chitosan derived from a crustacean’s shell was employed to remove hazardous metal ions from wastewater [135]. Due of the presence of amino groups in the chitosan sample, the amount of adsorption increased with the degree of deacetylation. The amount of adsorption was increased with the degree of deacetylation of chitosan because of the presence of amino groups in the chitosan sample. The molecular imprinting method was used to modify the chitosan by the use of methacrylic acid. This modified adsorbent was used for the extraction of Cr^6+^ ions from the contaminated Xiangjiang river water. It was found that imprinted polymer has more vacant sites available and adsorption value increased after modification. The adsorption equilibrium was obtained in eight hours within pH value 4.5−7.5. The maximal sorption at equilibrium shown by imprinted chitosan was 98.3%. The highest adsorption value obtained from Freundlich and Langmuir adsorption isotherms was 15.784 mg/g [136]. Suresh Kumar et al., have synthesised chitosan-magnetite nanocomposite strips for the removal of the heavy metal solution containing Cr (VI) ions. They have proposed a mechanism of adsorption and chromium removal as shown in Figure 5 [137].

**Figure 6:**
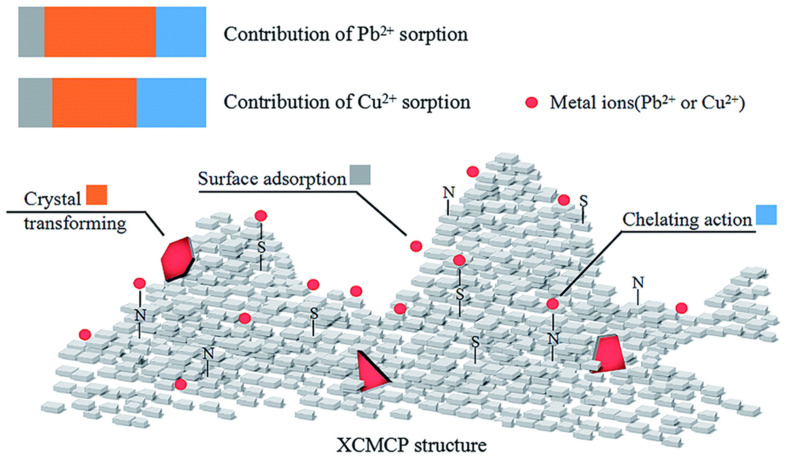
Adsorption mechanism diagram and contributions of adsorption Pb^2+^ and Cu^2+^ onto XCMCP [138].

The time to attain equilibrium by chitin was six hours (5.33 g) for the Zn^2+^, four hours for Cd^2+^ (13.37 mg/g), and three hours for Cu^2+^ (20.5 mg/g). In case of chitosan, equilibrium was achieved within five hours for Zn^2+^ (19.31 mg/g), four hours for Cd^2+^ (37.7 mg/g) and two hours for Cu^2+^ (44.7 mg/g) [137]. Lv et al., synthesised xanthate crosslinked with chitosan/PVA for the extraction of Pb^2+^, Cu^2+^ from an aqueous solution. This adsorbent can be used several times and effectively because of the presence of nitrogen, sulphur atoms. These atoms present on xanthate crosslinked with chitosan/PVA (XCMCP) can effectively coordinate with toxic metal ions Figure 6. The batch method was used to study adsorption phenomena by using 150 mg of dried XCMCP for different concentrations of metal ions solutions [138].

#### 6.6.4. Seaweed and Alginate

Seaweed contains a large amount of metal-sorbing biomass. Some seaweed having polysaccharide content was recognised for the ion exchange properties. These properties were especially noticeable in brown algae and the highest sorption amount was found to be about 67 mg of cadmium per gram of seaweed [109]. Furthermore, *Ulva lactuca* green algae were less effective in comparison to brown seaweeds for adsorption of cadmium and mercury removal. Even though seaweed has a high sorption capacity, they have a property to break down materials as well, and therefore they were modified through crosslinking with glutaraldehyde, formaldehyde, and polyethyleneimine [110]. In algae, crosslinking can enhance the firmness and mechanical properties of the algae. *A. nodosum* was found to adsorb 215 mg cadmium per gram of raw seaweed and 149 mg cadmium per gram of formaldehyde-crosslinked seaweed. A. nodosum was more viable as compared to other seaweeds tested. Leusch et al., prepared different sizes of two marine algae, *Sargassum fluitans* and *Ascophyllum nodosum*, for the extraction of toxic metal ions like cadmium, copper, lead, nickel, and zinc [139]. Alginate, a natural biopolymer, is also utilised as an adsorbent to remove metals including Pb, Ni, Hg, and Cu, as well as organic contaminants, from wastewater. Alginate is a structural component of marine brown algae such as *Macrocystis pyrifera*, and it is abundant in nature. Because of its electrostatic interactions, this polysaccharide has several anionic or cationic groups in its structure, giving it distinctive physical features. Alginate is made up of 1,4-linked D-mannuronic and L-guluronic acids. Alginate is an anionic polymer found in the outer cell walls of brown algae such as kelp. Alginate’s major component is alginic acid, whereas sodium alginate (SA) is the sodium salt of alginic acid, having numerous free hydroxyl and carboxyl groups scattered along the polymer’s backbone chain. When Ca^2+^ is added to the SA solution, it displaces some of the H^+^ and Na^+^, resulting in the formation of calcium alginate (CA) gel. The capacity of sodium alginate to bind to multivalent cations follows the following sequence: Pb^2+^ > Cu^2+^ > Cd^2+^ > Ba^2+^> Sr^2+^ > Ca^2+^ > Co^2+^ > Ni^2+^ > Zn^2+^ > Mn^2+^ [140].

Hassan et al. investigated As removal using three different adsorbent materials: KOH-activated carbon-based apricot stone (C), calcium alginate beads (G), and calcium alginate/activated carbon composite beads (C) (GC). GC showed the maximum As (V) adsorption (66.7 mg/g at 30 °C), according to the data [141]. To absorb Cr (VI) and As (V) from wastewater, Vu et al. created magnetite GO encapsulated in calcium alginate beads (mGO/beads) [142]. Soltani et al. entrapped silica nanopowders within calcium alginate and found that a pH of 5.0 was best for Pb (II) adsorption, with a maximum adsorption capacity of 83.33 mg/g [143]. For example, Papageorgiou et al. found that Ca-alginate beads with numerous oxygen-enriched functional groups (–COOH/–OH) had a reasonably high capture capacity for Pb^2+^ (360.11 mg/g) [144].

#### 6.6.5. Xanthate

Sulphides, thiols, dithiocarbamates, dithiophosphate, and xanthates are sulphur-containing compounds. Xanthates are well known because they are obtained through the simple reaction of an organic hydroxyl-containing substrate with carbon disulphide. These reagents are economical and synthesise through easy organic reactions [18]. In the case of xanthate, an ion-exchange reaction was used to remove toxic metal ions. Both cellulose and starch xanthate was effective in the removal of metal ions of cadmium (19.9, 33.3 mg/g) and chromium (19.7, 17.6 mg/g) with good adsorption capacities. Mercury, however, was poorly adsorbed (0.64 and 1.15 mg/g) on the above matrices [111]. Flynn et al. reported xanthate sawdust for the sorption of toxic metals. The adsorption capacity of xanthate sawdust was reported for cadmium by the batch method in the range of 15.7–21.4 mg Cd/g. For different metals ions like manganese, cobalt, nickel, silver, zinc, mercury, and lead sorption values were found in the range from 0.3 to 0.4 mg/g [112]. Chakraborty et al., compared insoluble baggasse (IBX) and insoluble wood xanthate (IWX). Maximum copper loading achieved with IBX and IWX were 26.9 ± 0.15 and 27.8 ± 0.39 mg/g, respectively, at xanthate dose of 0.125 g/L. [145].

#### 6.6.6. Zeolites

**Figure 7:**
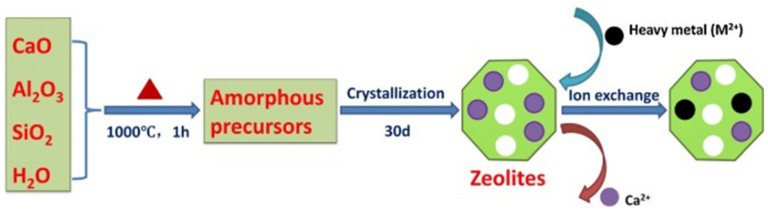
The mechanism of Cd (II) and Pb (II) ions adsorption in montmorillonite and zeolites by ion exchange method. Adapted with permission from Reference [149].

Zeolites are obtained from nature through silicate minerals and can be prepared by artificial methods. Zeolites are able to adsorb different sizes, shapes, and polarity adsorbates. Hence, zeolite is also known as a molecular sieve. Zeolites (clinoptilolite) showed strong attraction for lead and other toxic metals [18]. Zeolites were considered better adsorbents because of their ion exchangeable groups. Zeolites are three-dimensional aluminosilicates of potassium, calcium, and sodium arranged in a tetrahedral structure where aluminum and silicon are surrounded by oxygen atoms. These are basically used in the ion exchange process as well as adsorbents to adsorb different metals and impurities. Leppert showed that the total adsorption value for zeolites is approximately 155.4 mg of lead per gram of zeolite [113]. Santiago et al. reported zeolites that were obtained by the reactions of organic cations ethylhexadecyldimethylaminonium (EHDDMA) and cetylpyridinium (CETYL) for the removal of metal ions. The obtained zeolite contained positively charged ions which were used to bind chromate ions as well as in the anion exchange process. Results demonstrated that cetylpyridinium modified zeolite executes better performance than ethyl hexadecyl dimethyl ammonium. Extraction values were found to be 0.65 mg of chromium per gram of zeolite with cetylpyridinium and 0.42 mg of chromium per gram of zeolite with ethyl hexadecyl dimethyl ammonium group. Hence, a clinoptilolite type of material and zeolite appeared to be powerful and economical for the extraction of toxic metals [146]. He et al. synthesised zeolites by the use of coal fly ash for the extraction of heavy metals like lead, cadmium, copper, nickel, and manganese from the aquatic system. Zeolite dose 1.0 g/L was used for extraction of single metal as well as multiple heavy metals having starting concentration of 100 ppm. The kinetics of the adsorption was also studied by mixing the zeolite in single-heavy metal and multi-heavy metal systems with the initial concentration of 100 mg/L. It was found that with an increase in adsorbent dose, removal efficiency gets increased. The results obtained from the experiments were in agreement with the Langmuir adsorption isotherm and the highest sorption value obtained for different metal ions Pb (II), Cu (II), Cd (II), Ni (II), and Mn (II) were 65.75, 56.06, 52.12, 34.40, and 30.89 mg/g and 45.28, 32.86, 26.93, 16.25, 14.63 mg/g for single and multi-metal ions. Sorption of different metals on the adsorbent followed the trend Pb (II) > Cu (II) > Cd (II) > Ni (II) > Mn (II) [147]. Natural zeolites were synthesised by Erdem et al., for the extraction of Co (II), Cu (II), Zn (II), and Mn (II) from wastewater. The batch process was used for the removal of metal solutions having a concentration range from 100–400 ppm and the dose of adsorbent used was 10 g for 500 mL metal ions solutions. The adsorption capacity of different metal ions on the adsorbent was decreased with an increase in the concentration of metal ions solutions. In zeolites, both adsorptions, as well as ion-exchange phenomena were used for the extraction. The order of the exchange of metals was found in the following sequence Co (II) > Cu (II) > Zn (II) > Mn (II). The highest exchange value was found to be Cu (II)-66.10%, Co (II)-77.96%, Zn (II)-45.96%, and Mn (II)-9.84% [148]. Chen et al. reported the use of synthetic mineral adsorbent for the removal of Cd (II) and Pb (II) ions from aqueous solutions by an ion-exchange method. They had shown that the adsorption process was pH-dependent and was found to follow the pseudo-second-order kinetic model and Freundlich isotherm model. They had explained the mechanism of Cd (II) and Pb (II) ions adsorption with the help of a schematic diagram Figure 7 [149].

#### 6.6.7. Clay

The negative charge on clay is due to the morphology of fine silicate which results in better adsorption values of clay. This anionic charge is balanced by positively charged species on the surface of clay and clay was able to adsorb cations, such as toxic metals. The expansive surface area of clay (up to 800 m^2^/g) increases the extraction capacity of clay [18]. Clay is found in three fundamental classes namely, kaolinite, micas (for example, illite), and semectites (for instance montmorillonite). Of the three species, montmorillonite clay has the smallest size of the crystal, more surface area, and maximum cation exchangeability. In this way, montmorillonite clay must possess a prominent adsorption capacity. Griffin et al. found that the elimination of mercury by montmorillonite was more prominent as compared to kaolinite [150]. Sharma et al., utilised clay wollastonite during his batch experiments. According to Langmuir isotherm, the maximal adsorption value shown by Wollastonite was 6.52 mg/g for nickel [151].

Chaturvedi et al. examined the sorption of chromium onto a 1:1 mixture of fly ash and wollastonite [152]. Oxides in the fly ash and wollastonite were responsible for the mixture’s sorption potential. Chromium was sorbed as an anion of chromium (HCrO_2_^−4^) and for that, a cation was needed to attract the metal ions. Above pH 2.5, alumina, SiO_2_ and CaO are negatively charge and maximum sorption took place at this pH. The extraction of negatively charged ions is more successful in strongly acidic conditions. Therefore, clay might be improved to enhance its adsorption capacity. Cadena et al. utilised bentonite, modified by substituting the natural exchangeable cations tetramethyl ammonium ion (TMA) to extract lead, chromium and the tetramethyl ammonium ions attached to resin has enhanced the clay sorption ability. It was found that 1 g of natural benotonic has an adsorption capacity of 6 mg for Pb^2+^ ions and 55 mg for Cr^6+^ ions, while tailored bentonic was adsorbed approximately 58 mg of lead ions and 57 mg of chromium ions [18]. Pradas et al. analyzed that acid treatment of modified natural bentonite decreases the sorption capacity of Cd and Zn, while high temperature treatment basically increased the sorption value whether bentonite was present in modified for or in the natural state [153].

#### 6.6.8. Ion Exchange Resins

**Figure 8:**
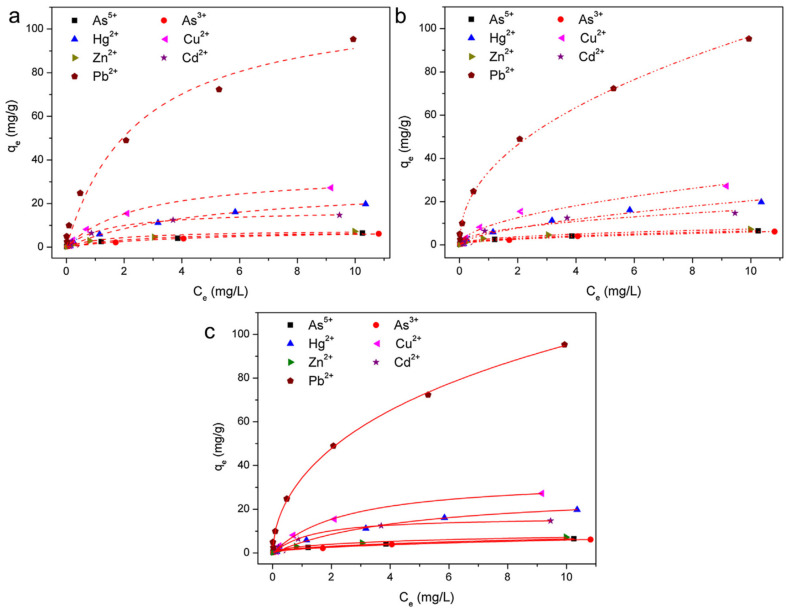
Adsorption isotherm for metal ions adsorption by cyshtcc-Fe_3_O_4_, (**a**): Langmuir fit; (**b**): Freundlich fit; (**c**): Sips fit. Reused with permission from [155].

The resins derived from polyhydric phenol and formaldehyde (HCHO) were cation exchange resins, and anion exchange resins were obtained from m-phenylene diamine, HCHO. These resins were exchanged with ions in solution with other ions of similar charges by an ion-exchange method in which ions in the solution were replaced with ions in the polymer matrix. Cationic exchange resins were mainly responsible for the removal of the cationic hardness of the water (Ca^2+^, Mg^2+^). However, chemically these were not very stable and tended to dissolve in basic solution. This prompted the quest for new ion exchange materials and cross-linked styrene-divinylbenzene resins have been produced [154]. These tars have the high synthetic and physical stability form of small beads/spheres. A further sulphonated group was attached to the styrene-DVB skeleton by reacting it with fuming sulphuric acid or chlorosulfonic acid. Sulphonated styrene-DVB resins have a higher exchange capacity at all pH and higher chemical stability, synthesis of their styrene-divinylbenzene copolymer matrices, anion, and cation exchange membranes have been patented by a number of organisations in the USA and UK. Ion exchange resin containing –COOH groups has been synthesised by the reaction of methacrylic acid with crosslinking agents and patented by numerous examiners. It is naturally opaque in appearance, greatly impervious to osmotic shock, and demonstrates no breakage on rehashed swelling (approximately 100%). Examinations have been undertaken in order to remove follow trace metal particles Ca, Mg, Cu, Zn, Fe, Mn, and purified water might be used for developing plants and in commercial enterprises. Styrene-DVB-based amber lite XAD resins were extremely prevalent for the extraction of organic impurities and color bodies from wastewater. Sulphonated styrene–DVB-based ion exchange resins were also additionally utilised for the extraction of poisonous metal particles. For the adsorption isotherm studies, the tested conditions were as follows: initial ion concentrations 0.5–20 mg/L, adsorbent dosage 1.5 g/L, temperature 30 °C, and pH 7. Cyshtcc-Fe_3_O_4_ had relatively high adsorption capacities for all the tested ions, especially for Pb2+ (235.63 mg/g), As (III) (66.3 mg/g) and As (V) (66.7 mg/g). Lead ions showed a high sorption capacity among the other metal ions for the resultant adsorbent (Figure 8) [155].

A bio ion exchanger was synthesised by using medicinal waste herb chicory for the extraction of lead and cadmium from water [156]. This herb chicory was then treated with calcium chloride to enhance the efficiency of the cation exchanger as well as the capacity of Pb (II) and Cd (II) ions removal. The utmost sorption efficiencies were found to be 103.1 and 53.8 mg/g for raw chicory waste and sorption by functionalised chicory was increased to 123.5 and 64.5 mg/g for the respective metal ions i.e., Pb (II) and Cd (II). Regeneration of water and heavy metal ions was undertaken by calcium chloride, sodium chloride, and nitric acid. A new nanosized ultrafiltration membrane was prepared by polyacrylonitrile as well as goethite nanoparticles. This membrane was used for the extraction of Cu (II) ions from aqueous solutions. Firstly, goethite nanoparticles having a diameter of 50 nm were synthesised. In the next step, these nanoparticles were embedded in the nanocomposite membrane. Response surface methodology based on Box-Behnken design was used to optimise the compositions of membranes. The impact of membrane composition on the extraction rate of the metal from the water was reviewed under constant parameters. The working parameters for the removal of metal ions and water penetration were 15%, 1.3%, and 1.0% for polyacrylonitrile, polyvinylpyrrolidone, and nanoparticle loading. The maximum copper ions removal was found to be 49.1% by hydrous metal oxide that contained polyacrylonitrile-based nanocomposite membranes [157]. Nanofiber-based ion exchange membrane was synthesised by the grafting of poly(acrylic acid) and poly(itaconic acid) to cellulose nanofiber mats [158]. Cadmium showed great attraction to poly (itaconic grafted acid) cellulose nanofiber mats in comparison to poly(acrylic acid) grafted cellulose nanofiber mats. PIA-based membranes were able to remove 220 mg Cd/g in comparison to traditional membranes. Highly porous styrene/2-ethyl hexyl acrylate/divinylbenzene was synthesised to produce polymerised high internal phase emulsion (ST/2EHA/DVB polyHIPE) solid foams strengthened by the use of the different quantities of silica nanoparticles through emulsion templating [159]. The sulfonic group was introduced in the resultant ion exchange membrane by the use of sulphuric acid as a functional group. The adsorbent amount of 0.25 mg per 100 mL solution and pH value equal to 6 were considered to investigate the adsorption kinetics of nickel ions. It was found that an increase in the amount of silica up to 3 wt.% increases the efficiency of the ion exchange membrane from 3.6 to 3.9 meq/g and also the introduction of the sulphonic group increases the extraction of nickel ions. Murray et al., synthesised sub-micro size polymeric ion-exchange resin (SMR) for the extraction of Pb^2+^, Cu^2+^, Zn^2+^, and Ni^2+^ from the aquatic systems along with natural organic matter. The river water and wastewater samples were tested with 153 varying concentrations of SMR particles (5, 50, and 500 mg/L). These ion-exchange resins were able to extract 82% of Pb, 46% of Cu, 55% ± 20% of Zn, and 17% ± 2% of Ni from stream water spiked with 500 μg/L of every metal. Likewise, in devastating water, the polymeric ion-exchange resin was found to expel 86% ± 0.1% of Pb, 38% ± 0.8% of Cu, 28% ± 1% 22 of Zn, and 11% ± 1% of Ni. The resin was able to remove 10% or less organic matter from stream water and less than 4% from wastewater [160].

### 6.7. Synthetic Polymer Adsorbents

**Figure 9:**
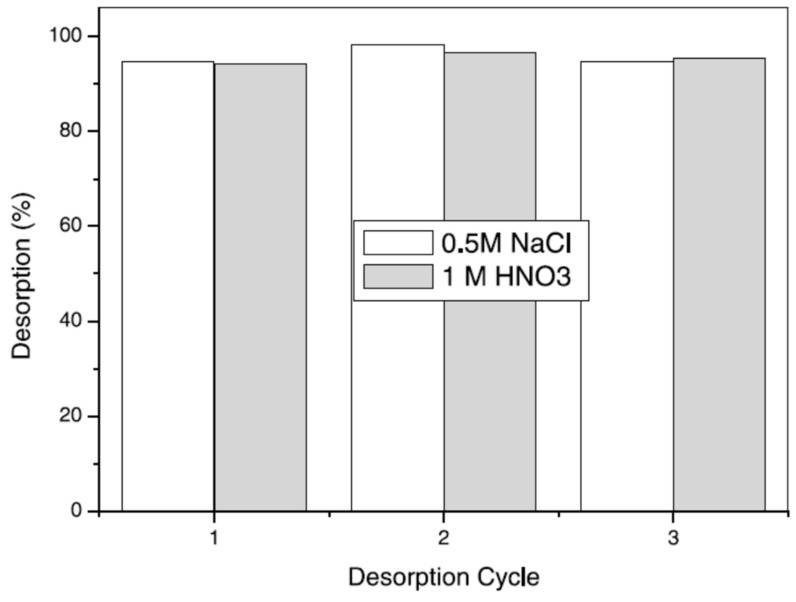
Desorption cycles of the poly (EGDMA-co-AAm) beads for Pb (II) metal ion. Reused with permission from [100].

There are a large number of synthetic adsorbents synthesised for different heavy metal ions removal and the sorption values of few adsorbents are presented in Table 3. Because of improvements in the field of polymer, new classes of adsorbents have come up, specifically the synthetic permeable polymeric adsorbents which have proper porosity, sufficient surface area, better dimensional stability, and a fast rate of adsorption. Polymeric adsorbents were permeable, porous spherical beads taking into account crosslinked poly(styrene-DVB) or phenol-formaldehyde condensate polymers [154]. Their high internal surface areas can sorb and desorb different types of ions dependent on the functionality and structure of the adsorbent. Polymeric adsorbents might have a few points of interest, including: (a) they can be easily available in a variety of shapes, for example, beads, membranes and fibers, (b) the surface can be changed by different techniques such as polymer brush arrangement, functionalisation, and molecular imprinting to enhance the removal ability of adsorbates, (c) specific sorption can be accomplished by means of functionalising the polymer substrate with the desirable ligands that have selectivity. They are likewise used for the extraction of poisonous toxic metals from contaminated water. Styrene-DVB copolymer-based macro reticular materials were studied by copolymerising styrene and DVB in the presence of organic diluents that result in polymers with high surface area and high porosity [161]. Furthermore, these beads have been functionalised and a new class of cation exchange resins with remarkable metal ion exchange capacity has been synthesised. Crosslinked permeable copolymers of methacrylic acid–N, N’ methylene bis acrylamide or ethylene glycol dimethacrylates were reported in the literature [162,163]. Polymethacrylic copolymers were assessed for the sorption of Ni and Cu ions from spiked metal particle arrangement in static and dynamic conditions. Poly (ethylene glycol dimethacrylate–N-vinyl imidazole) beads for the extraction of toxic metal ions have additionally been reported by Kara et al. [26]. The uptake capacity was found to be Hg > Cd > Pb and the sorption value was 45.6 milligram for cadmium, 92.5 mg for lead per gram of resin individually. Kesenci et. al, synthesised poly (ethylene glycol dimethacrylate-acrylamide) copolymer for extraction of (lead mercury and cadmium) metal particles in aqueous solution, and metal uptake results were observed to be in the order lead > cadmium> mercury [100]. They have also checked the desorption capacities, which was found to be up to 98%, and observed that it did not prominently alter during the repeated cycles (Figure 9). It has also been observed that the desorption tests were not affected by the type of eluent.

Etorki and Walli reported water dissolvable polyvinyl pyrrolidinone (PVP) for extraction and pre-concentration of Hg (II) ions. PVP contains a pendant lactam ring having oxygen and nitrogen groups which was used to bind toxic metal ions [164]. Kavakh et al., researched the sorption characteristics of metal ions by poly(N,N-dimethyl aminoethyl methacrylate) hydrogels. The sorption value of toxic ions by hydrogel was found in the order Cu > Zn = Co > Pb >> Ni > Cd and all gel systems followed the Langmuir adsorption isotherm [170]. Polypyrrole was utilised for the removal of arsenic from wastewater as reported by Eisazadeh et al. [171]. Arsenic was removed from the aquatic system by the diethylenetriamine functionalised copolymer glycidyl methacrylate (GMA) on chloromethylated cross-linked styrene-divinylbenzene, synthesised through the atom transfer radical polymerisation technique on the surface of the polymer. The amine group was introduced through the reaction of an amino moiety of diethylenetriamine and epoxy group of GMA. 0.1 g amine-functionalised polymer was used for the extraction of 7 mmol/L As^5+^ solutions at various pH values and the mixture was agitated at a speed of 200 rpm for 12 h. Small amounts of metal ions solution around 1.0 ml were taken from the mixture to study the adsorbed concentration. A mixture of arsenic ions 7.0 millimole per liter and other metal ions such as Na^+^, K^+^, Ca^2+^, Mg^2+^, Fe^3+^ and Zn^2+^ (7 mmol/L) were taken to check the selectivity of 0.1 g of diethylenetriamine functionalised adsorbent for As^5+^ over the other ions. The adsorption process from the mixture was explained through the hard-soft acid-base concept. The metal ions like Na^+^, K^+^, Ca^2+^, Mg^2+^ are hard acids while Fe^3+^, Zn^2+^ are borderline acids. Fe^3+^, Zn^2+^ ions being soft acid can interfere with As^5+^ in the adsorption process. However, it was found that DETA functionalised polymer effectively adsorb arsenic than iron, zinc metal ions. The adsorption capacity of As^5+^ was reached around 5.25 mmol g^−1^ at pH 4.0 follow the Langmuir adsorption and pseudo-second-order kinetic equation [172]. (E)-2-[(1H-Imidazole-4-yl)methylidene]-hydrazinecarbothioamide ligand (EIMH) synthesised by the reaction of thiosemicarbazide and imidazolecarboxaldehyde by using a mixture of ethanol and water in the presence of sulphuric acid as catalysts used to remove heavy metal ions such as lead, copper, and cadmium ions from wastewater. The highest uptake occurred within 10 min with sorption efficiency of 99.80% of Pb (II), 99.25% of Cu (II), and 98.68% of Cd (II) were removed at pH 2–8 [173]. Various polymeric material-based adsorbents used for removal of toxic metal ions is given in Table 3.

Chelating resins are ion exchange resins that are synthesised to bind anion or groups of ions. Chelating resins contains donor groups with lone pair of electrons such as nitrogen, oxygen, sulphur and phosphorous. These moieties are able to form a coordinate bond with metal ions selectively. Kim and Maeng, synthesised poly(acrylamidoxime) chelate resins by redox polymerisation of AN and DVB followed by reaction with hydroxylamine hydrochloride. Complexation properties of resin were observed to be viable for sequestering of La, Ce, and Nd from aqueous solutions [174]. Coutinho et al. additionally reported the complexation ability of copper ions by utilising chelating resin containing amidoxime groups [28]. Liu and Sun investigated chelating resins containing a cysteine group by reaction of hydrolyzed macroporous polyacrylonitrile copolymers with L-cysteine and 1,6 hexanediol. This resin was very particular for silver, mercury, gold, and platinum in aqueous acidic solutions [175]. Trochimczuk reported a novel chelating polymer with an enhanced extraction capacity of metal ions [176]. These copolymers were prepared by the functionalisation of a vinyl benzyl chloride–divinylbenzene copolymer with the sodium salt of diethyl malonate after that reaching the modified resin with amine i.e., ethylene diamines, diethylenetriamine, 2-aminomethyl pyridine, 3-aminopropyl imidazole. The chelating properties of the synthesised resins were studied by the formation of an acetate buffer of pH 3.7 and 5 for copper, cadmium, nickel, and zinc metal ions. Sorption of the following cations on the improved resin was superior to the adsorption on the original polymer containing N-substituted amides of monocarboxylic acid. New chelating hydrogels were synthesised by the reaction of N-vinyl imidazole and acrylonitrile [177]. These gels were used in heavy metal ion adsorptions which were synthesised by irradiation of binary monomer mixture followed by reaction with hydroxylamine hydrochloride.

In another study, poly(hydroxyethyl methacrylate) (PHEMA) beads containing thiazolidine group (0.318 mmol/g) were prepared by Saglam et al. and used for the elimination of Pb^2+^ and Cd^2+^ metal ions from synthetic solution in different acidic conditions (pH = 0.3–7.0). Adsorptions of Pb^2+^ and Cd^2+^ onto polymer microbeads were 0.336 and 0.397 mmol/g correspondingly. However, in the case of a mixture, the polymer was more selective for Pb ions. PHEMA microbeads having thiazolidine as functionality can be regenerated by washing with HCl acid (0.05 M). The obtained resins can be used repetitively with the same adsorption capacity [178]. Qu and co-workers reported chelating resin synthesised by crosslinked polystyrene functionalised with 2, 5 dimercapto-1, 3, 4-thiadiazole for the elimination of mercury ions [105], and adsorption results were explained by the Langmuir adsorption isotherm model. Regeneration of chelating resins could be undertaken by 2% thiourea in 0.1 mol/L HCl. [179].

The sorption behavior of amidoxime poly(N,N’-dipropionitrileacylamide) [Poly(DPAAm) and non-woven fabric grafted with the same UO_2_^2+^, Pb^2+^, Cu^2+^, and Co^2+^ at high concentrations were investigated for metal ion adsorption studies [180]. Particulate amidoximated poly(DPAAm) has shown a better sorption value in comparison to the amidoximated non-woven fabrics for all toxic ions mainly for UO_2_^2+^ ions. Sorption values were obtained such as 400 mg UO_2_^2+^/g of dry amidoximated poly (DPAAm) and 250 mg UO_2_^2+^/g of dry amidoximated graft polymer. Chettiar and Sreekumar synthesised dithiosemicarbazone functionalised polymer, namely, poly(styrene-divinylbenzene) and poly (hydroxyethyl methacrylate-divinyl benzene) beads having definite porosity for adsorption of toxic metal ions [181]. Improved crosslinked polyacrylamides containing various functional moieties have been synthesised by transamidation reaction in the aquatic and non-aquatic medium by Hofmann’s reaction. These functionalised resins were used for the elimination of copper, cadmium, and lead ions from synthetic solutions at various pH [182]. The metal ion adsorption value was obtained at around 0.11–1.71 mmol/g of polymer. The above-synthesised polymer containing secondary amine moieties was more specific for copper ions (99.4%), while the polymer with secondary amide and carboxylate moiety was more selective towards lead ions (99.5%). With a decrease in the pH of the solution, the resin’s specificity for copper ions decreased while it increased for lead ions.

Jesus et al. reported macroporous poly(4-vinlypridine–DVB) with a large surface area (130 m^2^/g) was synthesised by suspension copolymerisation of 4-vinyl pyridine and divinylbenzene. This resin has a pyridine group that was able to adsorb 94% chromium ion at pH 6.5 [183]. Cardoso and co-workers also removed 95% of chromium from wastewater solution having concentration 4–500 ppm by using poly(4-vinyl pyridine-DVB) copolymers, functionalised with N-oxide groups [184]. Poly(acrylamide-4 vinyl pyridine) was investigated by Hamshary and co-workers to adsorb metal ions namely Mn^2+^, Co^2+^, Ni^2+^, Cu^2+^, and Zn^2+^ [185]. The resin showed more attraction for Cu^2+^, Ni^2+^ and extraction ability of metal ion was in the subsequent pattern Ni^2+^ > Cu^2+^ > Zn^2+^ > Co^2+^ > Mn^2+^. The removal ability of metal ions by resin was decreased as the pH of the solution was decreased. Denizli et al. studied the sorption performance of poly (2-hydroxy ethyl methacrylate-methacrylolmidophenylalanine) poly (2 HEMA-MAPA) beads [186]. The reaction of methacrylolychloride with phenylalanine resulted in a metal complexing comonomer MAPA. The sorption values obtained for different metals ions by the above adsorbent were 669.4 mg/g for mercury, 584.4 mg/g lead, 268.4 mg/g for cadmium, 204 mg/g for arsenic, and 115.2 mg/g for chromium. Adsorption studies and regeneration of metals suggested that poly (2-hydroxy ethyl methacrylate-methacrylolmidophenylalanine) was a superior metal sorbent that had a large possibility for environmental safety.

Denizli studied the removal of lead ions by using poly (hydroxyethyl methacrylate–N-methacryloyl–L-glutamic acid) [165]. The sorption of lead ions by the adsorbent PHEMA was insignificant (0.38 mg/g) and increased to 348 mg/g after the incorporation of the chelating group on the surface of poly 2-hydroxy ethyl methacrylate. The sorption values were found to be 42.5 mg/g for lead, 26.8 mg/g for mercury, and 17.6 mg/g for cadmium at 0.5 mol/L metal solution concentrations. Poly(acrylamidoxime) chelating fiber was prepared by the reaction of polyacrylonitrile with hydroxylamine for extraction of copper and mercury ions as reported by Bilba et al. [187].

Hazer and Kartal reported a novel chelating polymer poly(acrylamidoxime–co {1-(2-pirydylazo} 2-naphthyl-2-methacrylate(APM) having three functional moieties such as amidoxime [C(NH_2_)=NOH)], azo(-N=N-) and carboxylic acid (-COOH) [188].The obtained polymer was able to extract trace amounts of uranium. The spectrophotometric method was used to determine U(VI) by considering arsenazo III as a complexing agent. Poly(acrylamidoxime–co {1-(2-pirydylazo} 2-napthyl-2-methacrylate (APM) resin was found to adsorb 24.2 mg of uranium per gram of resin. Poly (ethylene glycolmethacrylate-vinyl imidazole) (PEGMA-VIM) was also used for the removal of Cu2+ metal ions [189]. The maximum sorption value for metal was 30 mg of copper per gram of polymer with an initial copper metal concentration of 400 mg/L under determined optimum conditions. Jing et al., prepared N,N’-di (carboxymethyl) dithiocarbamate) chelating resin for the removal of toxic ions from devastating streams [190]. The chelating resin prepared by reaction of N, N’ (carboxymethyl) dithiocarbamate with chloromethylated PS-DVB matrix was used for the sorption of heavy metal ions. Poly(4-(4vinylbenzyloxy)2-hydrobenzaldehyde P(VBH) a chelating polymer was able to remove ppb level cadmium from the aquatic system [191]. The sorption data follow up the Langmuir isotherm represented the unilayer coverage of the heavy metal on the resin surface. Denizli reported a new adsorbent synthesised by suspension copolymerisation method i.e., alkali blue 6B attached poly(EGDMA-HEMA) microbeads were used for the extraction of metal ions [192]. Adsorption of cadmium, copper, zinc and lead ions from the synthetic mixture of ions was studied in the concentration range 1–500 ppm at different pH values (1.5–7.5). The highest adsorption of ions onto alkali blue 6B the attached resin was 5.5 mg/g for Cd, 2.3 mg/g for Cu, 41.4 mg/g for Zn, and 125 mg/g for Pb [193]. Kasgoz et al. reported the modified polyacrylamide hydrogels synthesised by transamidation and Hofmann reactions by utilising various compounds of amine [182]. The resultant resins were utilised for the elimination of Cu^2+^ ions at pH = 5.5 by the batch technique. The highest extraction value of resin was 2.93 mmol/g for copper obtained after a revival cycle. 

Arsalani and Hosseninzadeh reported EDTA functionalised polyacrylonitrile for the adsorption of metal ions. The maximum and minimum metal adsorption values were found to be 5.2 mmol/g for Ni^2+^ at pH 5.0 and 1.5 mmol/g for Co^2+^ at pH 1.0 respectively [194]. Jermakowicz-Bartkowiak et al. synthesised new functional resins by reacting poly (vinyl benzyl chloride-acrylonitrile-divinyl benzene) matrix with ethylenediamine, bis(aminopropyl) amine, bis(aminohexyl) triamine, tris(2-amino ethyl) amine. Amine functionalised resins with 2.8–5.0 mmol of the amino group per gram of any resin were treated with 5-ethylthiourea. Adsorption capacities of resin for gold, platinum, and palladium chloro complex were found to be 190, 245, and 280 mg/g, respectively [195]. The sorptions of metal onto the amidoxime improvised polyacrylonitrile (PAN) nanofibers were investigated by Saeed et al. [169]. The equilibrium adsorption values for Cu^2+^ and Pb^2+^ were 52.70 and 263.45 mg/g demonstrating unilayer sorption on the nanofiber. Memon et al. examined the extraction of cadmium (Cd) from aqueous media by using 2,2′ bipyridine modified polystyrene foam [196]. Maximum 90% sorption was obtained at pH 7 after 30 min of mixing of adsorbent with metal ions, solution and adsorbent can be regenerated with HNO_3_. Removal of nickel from wastewater using phenol-formaldehyde resin (Duolite XAD -761) with anionic surfactant sodium dioctyl sulphosuccinate and EDTA–disodium salt was reported [197]. The pseudo-second-order kinetic model was used to explain the sorption of Ni on modified XAD-761 resin. They have also reported extraction of lead (Pb) from synthetic solution by means of dioctyl sodium sulphosuccinate–EDTA tailored amberlite XAD-7 HP resin (acrylic ester-based resin [197]. Similarly, fluoride ions in drinking water were also removed by amine-modified amberlite XAD-4 poly (Styrene–DVB) based resin [198]. The uptake value of fluoride ions by the modified amberlite XAD-4 resin was 5.04×10–3 mol/g and took place through an ion exchange adsorption mechanism. Polypropylene fiber was grafted with acrylonitrile and then treated with diethylene triamine to enhance the sorption capacity of grafted polymer for mercury ions [166]. Aminated chelating fiber has a high adsorption capacity for Hg 657.9 mg/g and this was due to the strong soft–soft interaction between mercury and amine. The extraction isotherm indicated that novel foam presents a high affinity for Cd and Pb due to the presence of good extraction sites (S, N, and O) in PU foam material. Chloro methylated polystyrene modified with 2-mercapto benzothiazole as a sorbent was also reported [199]. The modified polymer has a maximum sorption capacity of around 0.493 mmol/g for Ag^+^ at pH 2 and desorption was effective with HNO_3_. Sun et al. reported new coordinating polymers by reacting ethylenediamine to crosslinked polystyrene through the sulphide linkage [200]. An adsorption study indicated that both the adsorbents had the best absorption capability for mercury, copper, and silver ions especially PSM-EDA which had more affinity for Hg ions. Among all the sorbents, amine-functionalised poly(glycidyl methacrylate)-based polymers had greater capacity to remove toxic metal ions and some of which are mentioned in Table 4. Haratake et al. investigated the triethylene tetra amine-functionalised poly (glycidyl methacrylate) sorbents for copper, zinc, cobalt, nickel ions sorption from seawater at the pH of water [201]. Atia et al. studied the removal capacity of copper and lead by ethylene diamine-functionalised poly (glycidyl methacrylate) sorbents and confirmed that sorption behavior depends upon the pH and surface properties of the sorbents [202]. Choi et al. researched poly (glycidyl methacrylate) sorbents with six different amine functional groups for palladium ions sorption and maximum removal of palladium occurred with diethylenetriamine, although desorption phenomena were not studied for palladium ions [203]. A study reported by Atia and co-workers on ethylene-diamine, diethylene-triamine, and tetraethylenepentamine functionalised magnetic PGMA resins for mercury ions extraction and maximum sorption values were enhanced with molecular chains [204].

Azanova et al. was the first to report the metal ion exchange capacity of the sulphonated glycidyl methacrylate-ethyleneglycoldimethacrylate copolymer [210]. It was observed that the reaction between sulphur trioxide monohydrate and GMA-EGDMA did not lose its ion exchange capacity even after 100 cycles. Glycidyl methacrylate type resins such as poly(glycidyl methacrylate-ethylene glycol dimethacrylate) have been discovered to be a suitable and versatile preliminary resource for the preparation of ion-exchange resins [211]. Amines react with epoxy moiety through ring-opening reaction and result in the formation of a polymer with suitable exchange kinetics for heavy metal ions because of the hydrophilic property of the obtained resin i.e., ring-opening produces an -OH moiety on the carbon atom β to the incoming amine [212].

A polymer having EDTA-type chelating units was able to adsorb heavy metal ions like iron, zinc, cadmium, lead, nickel, copper, and cobalt ions. The resin possesses a diethylene triamine tetra acetic acid group that was more efficient in the softening of water. Bicak et al. continued their work on the efficient removal of metal ions using polymer-supported pendant urea groups. This resin was prepared by reacting GMA-MMA-DVB resin with triethylenetetramine and subsequent reaction with acidic isocyanate [205]. The polymer obtained holds a urea moiety with a removal capacity of 7.8 mmol/g and showed a better affinity for mercury with a loading capacity > 6.7 mmol/g.

Bayramoglu and Arica reported poly (GMA-MMA-EGDMA) based polymeric beads containing a ferric oxide group [213]. These magnetic beads were used for the removal of Hg ions and the best possible uptake of mercury took place at pH 5.5. The highest sorption ability of mercury ions by using this resin was 124.8 mg/g. The resins containing divinylbenzene (DVB) as a crosslinker were immobilised with tetra ethylene pentamine, result in an amine functionality in the resins GMA/DVB/TEP (R1-en) and GMA/MBA/TEP (R2–en) correspondingly in the presence of suspended magnetic particles [214]. The removal ability of two resins was investigated for molybdate anions and sorption values were found to be 4.24 and 6.18 mmol/g for (R1-en) and (R2-en) adsorbents. Renewal ability up to 90–96% was obtained by the use of ammonia buffer [215]. The resultant resin was found to sorb Cu or Pb ions considerably. However, resin showed more affinity for copper over lead ions in the mixture of both metal solutions. The removal of cadmium ions from aqueous solution by sulphonated poly(glycidyl methacrylate) SPGMA was also studied [216]. The maximum sorption capacity of cadmium onto synthesised SPGMA was 555.55 mg/g. The sorption rate of hexavalent chromium ions onto amine-functionalised glycidyl methacrylate copolymer was studied by Nastasovic et al. [217]. Two samples of macroporous crosslinked poly(GMA-EGDMA) functionalised with ethylenediamine and diethylene triamine moieties having different pore sizes of the polymers were prepared through the suspension copolymerisation method. The rate of sorption of chromium by amino-functionalised poly (GMA-EGDMA) was studied individually for each ion as well in a mixture of solutions. Kinetic models were used to explain the phenomena of metal sorption. Chen et al. reported cross-linked poly(glycidyl methacrylate–aspartic acid) was used for the recovery of Cu and Cd ions from aqueous solution by using chelating resin [207]. Equilibrium sorption values were found to be 1.40 and 1.28 mmol/g for Cu and Cd ions, respectively in non-competitive conditions. However, under competitive conditions, adsorption selectivity for Cu ions was observed in the presence of Cd at pH 2–2.5. Grafting of glycidyl methacrylate with dimethylamine and HCl leads to the formation of an anion exchanger bearing N^+^HR_2_ Cl^−^ functional group [218]. The resin was found to remove 99.6% vanadium metal ion in the pH range of 4–6. Gokila and co-workers prepared chitosan and alginate nanocomposites for the extraction of chromium (VI) from wastewater [219]. Chitosan and alginate nanoparticles were synthesised separately by an ionic crosslinking process by using sodium tripolyphosphate, calcium chloride, and its nanocomposites of ratio 1:1 were synthesised in the presence of glutaraldehyde as a crosslinker. An adsorption study has been done by the batch method and various factors have been studied such as the effect of rate of adsorption, the effect of initial concentration, adsorbent dose, pH, and stirring time. On studying the effect of time on adsorption phenomena, it was found that CS-AL nanocomposites with glutaraldehyde showed maximum adsorption within 300 min.

Liu et al. synthesised layer-by-layer (LbL) assembled forward osmosis (FO) membranes by accumulating several polyethyleneimines (PEI) and sodium alginate (SA) bilayers on polydopamine-functionalised polyvinylidene fluoride (PVDF) supportive membrane [220]. This membrane easily removed the heavy metal ions i.e., Cu(II), Ni(II), Pb(II), Zn(II), and Cd(II) from their synthetic solutions. The concentrations of these five heavy metal ions in the resulting solution were 1.0 g/L, 2.0 g/L, and 5.0 g/L, respectively. The removal of heavy metal ions by forwarding osmosis (FO) membranes was influenced by the pH of the synthetic solution, time, temperature, regeneration as well as concentration. Huang and co-workers synthesised permeable adsorbents of ZIF-8 and ZIF-67 and used them for the extraction of Pb^2+^, Cu^2+^ from wastewater [87]. Lam et al. studied the extractions of nickel ions from contaminated solutions by ultrafiltration membranes of natural chitosan or synthetic carboxymethyl cellulose through electrostatic interactions [221]. Here, both cellulose and carboxymethyl cellulose were mixed together for one hour at room temperature to obtain complete dissolution. The concentration of nickel ions ranged from 1 to 100 ppm corresponding to molar concentrations from 1.7 × 10^−5^ to 1.7 × 10^−2^ mol/L. Above mentioned, two polymers have the same removal tendency at neutral pH value; however, chitosan has proved outstanding elimination performances in basic conditions (above its pKa), whereas carboxymethyl cellulose has shown poor removal capacity at pH (3.5–4.0). However, CMC would be preferred to chitosan in natural conditions (4 < pH < 8) since its effect on the permeation flux was less important. Lignin xanthate resin (LXR) was prepared by a two-step process from alkaline lignin and CS_2_. According to the Langmuir model, the maximum amount adsorbed by this resin was 64.9 mg/g for lead ions at 30 ^o^C. Adsorption of lead ions by lignin xanthate resin (LXR) was more effective for lead ions than alkaline lignin due to the xanthate group. The adsorption capacity of lead ions was increased steadily to 62.5 mg/g with increasing pH from 1.0 to 5.0. The adsorption of Pb^2+^ on LXR achieved equilibrium within 90 min [222]. Ali and co-workers reported the nanoparticle-containing cellulose for the removal of heavy metal ions. Cellulose was impregnated with silver and zinc oxide nanoparticles by using the co-precipitation method [223]. It was found from the batch method that the adsorption of metal ions on zinc and silver NPs impregnated cellulose was very rapid and achieve equilibrium within 30 min. The cellulose impregnated zinc and silver composites adsorbents showed excellent metal affinity in the order of Hg^2+^ > Ni^2+^ > Cr^3+^ > Co^2+^. Adsorption capacities of cellulose impregnated with silver and zinc oxide nanoparticles was found to be Hg^2+^ = 104.6 mg/g, Cr^3+^ = 56.52 mg/g, Co^2+^ = 45.33, Ni^2+^ = 61.48 mg/g, Pb^2+^ = 37.21 mg/g and Hg = 789.3 mg/g, Cr^3+^ = 266.3 mg/g, Co^2+^ = 245.1 mg/g, Ni^2+^ = 222.9 mg/g, Pb = 92.22 mg/g for 1.0 g of sorbent amount. For 0.5 g sorbent amount of ZnCt, adsorption capacities were 107.8 for Hg^2+^, 83.02 for Cr^3+^, 50.44 for Co^2+^, 43.81 for Pb^2+^ and 101.2 mg/g for Ni^2+^ ion. For 0.5 g adsorbent dose of AgCt, adsorption capacities were 554.5 mg per gram for mercury, 157.75 mg/g for chromium, 114.1 for cobalt, 84.595 mg/g for lead, and 204.935 mg per gram for nickel ions calculated according to Langmuir adsorption isotherm.

**Figure 10:**
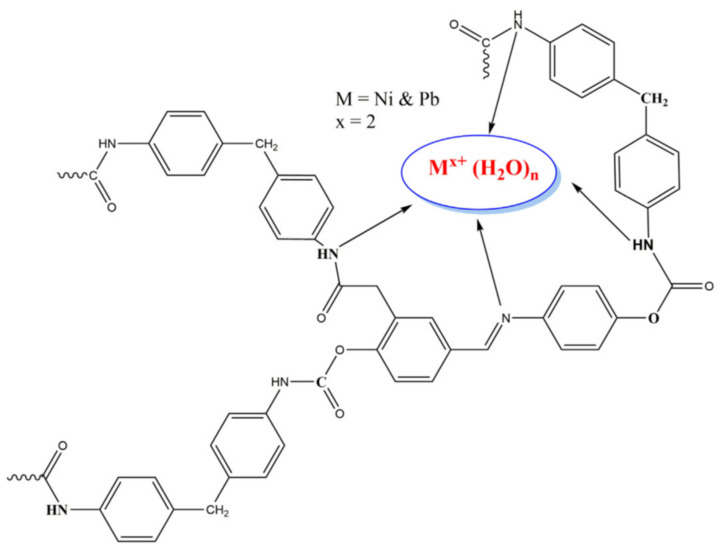
Adsorption mechanism of PU. Reused with permission from [167].

Zhou et al. synthesised a porous jute/polyacrylic acid gel by using free-radical polymerisation of acrylic acid in jute aqueous solution [224]. The Jute/PAA gel sorbent could proficiently extract particularly cadmium and lead toxic metal present in industrial effluent. Adsorption capacities of the jute/PAA gel sorbent were 401.7 mg/g for cadmium and 542.9 mg/g for lead toxic metal ions. Additionally, the sorption equilibrium achieved within ten minutes for 40 ppm of cadmium and lead by using 1.0 g/L sorbent. In the meantime, the extraction efficacies reached 81.0% for lead (C0 = 3.825 ppm), 79.3% for cadmium (C0 = 6.075 ppm), 83.4% for copper (C0 = 9.325 ppm), 29.8% for zinc (C0 = 188.6 ppm), 22.3% for manganese (C0 = 17.05 ppm), 96.2% for chromium (C0 = 0.25 ppm) and 99.8% for iron (C0 = 9.75 ppm) in melting wastewater using 1.0 g/L sorbent in two hours. Unique polyurethane (PU) was reported for the extraction of Pb^2+^ and Ni^2+^ ions from a synthetic mixture. The maximum amount of toxic metal ions adsorbed by polyurethane for Pb^2+^ and Ni^2+^ ions were obtained through the Langmuir isotherm was 236.5 and 217.5 mg/g, respectively. The adsorption competence of the polyurethane was retained even after five cycles [167]. The adsorption mechanism is shown in Figure 10 where a co-ordinate bond is created among the donor atoms of the binding sites of the polyurethane with steel ions. This behaviour is further verified in the desorption process.

Feng et al. reported porous attapulgite (ATP)/polyethersulfone beads were synthesised by the phase-inversion method [168]. The adsorption capacity of such beads was 25.3 mg/g for Cu (II) and 32.7 mg/g for Cd (II). Racho and co-workers reported modified nylon fibers for the removal of Pb^2+^ and Cr^6+^ having 93.8% and 25.6% adsorption capacities [225]. Their results showed a high capacity of the adsorbent (qmqx = 48 mg/g and 45 mg/g) for Pb^2+^ and Cr^6+^ at pH 5 and 3. It was also revealed that after functionalisation, the adsorption capacity of modified nylon fibers increased by 90% and 20% for Pb^2+^ and Cr^6+^, respectively. Argun et al. synthesised oak sawdust, naturally occurring materials as an adsorbent [226]. Sawdust was modified with an HCl solution appropriate for the extraction of toxic metals from metal solutions. This adsorbent mainly adsorbed three metals ions Cu^2+^, Ni^2+^, and Cr^6+^ of concentrations in the range 1 to 100 mg/L. According to the Langmuir adsorption model, sorption values were found to be 3.22, 3.29, 1.70 mg/g for Cu^2+^ Ni^2+^ and Cr^6+^ particularly. The maximum amount of Cu (II) ions were removed at pH 4.0(93%), while Ni (II) and Cr (VI) showed their maximum efficiencies (82%) at pH 8.0, (84%), and at pH 3.0 for chromium.

Chen et al. synthesised polyamine-type chelating resin by using the method of combining atom transfer radical polymerisation (ATRP) technology and hyperbranched polymer principle to synthesise the high-efficiency chelating resin [227]. This resin effectively removed As^5+^ and Cr^6+^ in acidic conditions while Cu^2+^, Pb^2+^, Cd^2+^, and Cr^3+^ were adsorbed effectively at high pH values. The highest sorption values of polyamine resin for As^5+^, Cr^6+^, Cu^2+^, Pb^2+^, Cd^2+^ and Cr^3+^ were 5.75, 4.44, 2.75, 1.40, 1.21 and 0.57 mmol/g, respectively. Gupta et al. reported amine-modified glycidyl methacrylate terpolymer for the extraction of mercury and lead ions [209]. The sorption effectiveness of Hg^2+^ and Pb^2+^ ions were determined by the use of the batch method. The highest uptake of lead ions was found to be 4.74, 4.76, and 4.73 mmol/g, and the highest mercury ions sorption was obtained at 4.76, 4.80, and 4.21 mmol/g respectively for PGMA–EDA, PGMA–DETA, and PGMA–TEPA polymers at their original pH. The sorption of mercury ions by the resulting resin was increased with increased time, and equilibrium was obtained within 20 h while the adsorption of lead ions by functionalised resins was achieved within 45 min.

**Figure 11:**
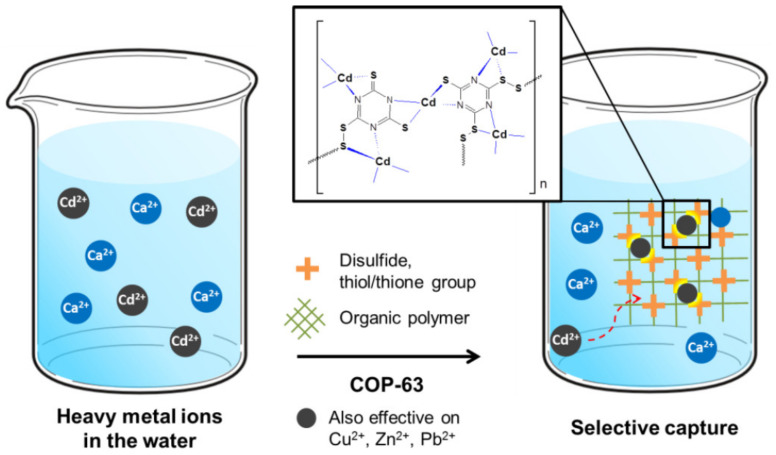
Selective interactions among heavy metal ions and sulphur groups in covalent organic polymer suspended in water. Reused with permission from [228].

Ko et al. reported disulphide functionalised covalently organic polymers (COP-63) for the extraction of toxic ions mainly Cd^2+^, Cu^2+^ and Zn^2+^ even in the presence of calcium ions from the aquatic system [228]. Figure 11 depicts the COP-63 sorption properties based on the hard-soft acid and base theory in which heavy metal ions in water are selectively engrossed in the organic polymer’s disulphide groups and dangling thiol/thione groups in the presence of competing cations. Pb^2+^ (99%), Cu^2+^ (97%), and Fe^3+^ (99%) were all removed by polyvinylamine at a concentration of 0.1 wt% [229]. Chen et al. reported a new chelating agent poly-ammonium dithiocarbamate (PADTC) and poly-sodium dithiocarbamate (PSDTC). For the extraction of copper, nickel, and zinc, different adsorption parameters were utilised, such as a 0.1 g adsorbent dosage, a 20-min reaction period, and a preliminary metal ion concentration of 100 mg/L [230]. Poly-ammonium dithiocarbamate had the highest adsorption capacity for Cu (II) 245.53 mg/g, Ni (II) 234.47 mg/g, and Zn (II) 226.76 mg/g.

### 6.8. Aerogels

Kistler used the term “aerogel” in 1932 to describe gels in which the liquid has been replaced by a gas [231]. The solid has a very low density, a large active surface area, and a low thermal conductivity. Aerogel is a substance that is made up of 99.8% air. Aerogels feature a porous solid network that contains air pockets, which take up the majority of the material’s interior space. The preparation of aerogels consists of three steps: sol-gel reactions, aging, and drying [232,233]. Carbon aerogels have recently piqued the interest of researchers due to their unique properties. Carbon aerogels can also be utilised to remove organic and inorganic contaminants from water/wastewater due to their unique qualities such as low density, three-dimensional (3D) interconnected porosity, high surface area, and extraordinary wettability [234]. The effect of carbon aerogel on the removal of Cd (II), Pb (II), Hg (II), Cu (II), Ni (II), Mn (II), and Zn (II) from aqueous solution was investigated by Meena et al. [235]. They found that heavy metal ion removal effectiveness is affected by concentration, pH, contact time, adsorbent dosage, and temperature. Their findings revealed that the maximum adsorption capacity of 400 mg/g for Cd (II) and 500 mg/g for Cu (II) was attained at an equilibrium time of 48 h, an optimal pH of 6.0, an adsorbent dosage of 10.0 g/L, a concentration of 3 mg/L, and a temperature range of 20 to 60 degrees celsius.

On the other hand, the effects of variable parameters such as agitation time, metal ions concentration, adsorbent dosage, and pH on Hg (II), Pb (II) removal from aqueous solutions were investigated by Goel et al. [236]. Kadirvelu and his co-workers studied equilibrium sorption in single, binary, and tertiary systems of three heavy metals Pb (II), Hg(II) and Cd(II). The maximum adsorption capacity, Q_0_ (mg/g), for adsorption of Pb (II), Hg(II), and Cd (II) on the CA in a mono-component system was 34.72, 34.9, and 15.53, respectively, according to the Langmuir isotherm. They used the Langmuir isotherm to simulate the adsorption behavior of metal ions in binary and ternary systems [Cd (II) + Pb (II)], [Pb (II) + Hg (II)], [Hg (II) + Cd (II)], and [Pb (II) + Hg (II) + Cd (II)]. They discovered that each metal’s maximum adsorption in binary and ternary systems is lower than in mono-component systems [237]. Goel et al. [238] employed carbon aerogel for the adsorptive removal of inorganic chemicals, particularly Cd (II) metal ions, in another work. The maximum adsorption capacity (Q_0_) determined for Cd (II) in the adsorption isotherm using the Langmuir equation was 15.53 mg/g. Microcrystalline cellulose was used as starting material for the preparation of cellulose-based aerogel using hydrothermal carbonisation. For the metal ions Cr (VI) and Pb (II), the adsorption capacity was determined to be 68 mg/g and 240 mg/g, respectively. Motahari et al. [239] employed resorcinol–formaldehyde (RF) aerogels modified with amine groups to adsorb Pb (II), Hg (II), and Cd (II) ions from aqueous solutions. Using the batch adsorption approach, they investigated the effects of contact time, solution pH, temperature, adsorption concentration, and initial metal concentration. After one hour, the highest adsorption capacity of metal ions Pb (II), Hg (II), and Cd (II) was discovered at pH 6, 6, and 5, respectively. For Hg (II), Pb (II), and Cd (II), the greatest value of the Langmuir maximum adsorption capacity (Q_max_) was determined to be 158.73 mg/g, 156.25 mg/g, and 151.52 mg/g, respectively.

Vesela et al. [240] studied the effect of pyrolytic temperature on the sorption ability of Cu(II) ions in carbon xerogels based on 3-aminophenolformaldehyde polymer. They provided a reasonable explanation for how pyrolytic temperature affects Cu (II) sorption. The sorption of A800 and A900 (for 800 and 900 C) was reduced due to a decrease in the number of nitrogen atoms in the pyridinic and pyridonic rings, as well as the positively charged surface of the samples, which repel positively charged Cu (II) ions. Zhan et al. [241] employed a viable green technique to study graphene/polydopamine modified multiwalled carbon nanotube (MWCNT-PDA) hybrid aerogels for effective heavy metal ions adsorption. They discovered that the hybrid aerogel exhibited a high adsorption capacity of 318.47 mg/g for Cu (II) and 350.87 mg/g for Pb(II) due to its appropriate active adsorption sites and interconnect porous structure. Han et al. [242] developed three-dimensional (3D) double network graphene oxide/polyacrylic acid (GO/PAA) hybrid aerogels under mild conditions utilising a one-pot in situ solution polymerisation technique that involved the polymerisation of AA as well as the self-assembly of functional GO sheets. The results of the GO/PAA aerogels demonstrated a high rate of Cu^2+^ ion adsorption, good recyclability, and a high adsorption capacity (390.34 mg/g). The Langmuir isotherm model characterised the adsorption equilibrium data well, and the Cu^2+^ ions adsorption kinetic data were well-fitted by the pseudo-second-order kinetic model. Pan et al. [243] published another GO hybrid investigation in which a porous calcium alginate/graphene oxide composite aerogel (mp-CA/GO) was used to evaluate the efficient removal of lead (Pb), copper (Cu), and cadmium (Cd) ions from water. Pb^2+^, Cu^2+^, and Cd^2+^ clearance rates were determined to be 95.4%, 81.2%, and 73.2%, respectively. Tabrizi et al. [244] described how graphene oxide aerogels (GOAs) were used to adsorb lead (II) (Pb (II)) metal ions from aqueous solutions. They used a unidirectional freeze-drying approach to create the aerogels from graphene oxide (GO) colloidal suspensions. Their findings revealed a high adsorbent capacity (q_mqx_ = 158 mg/g) and a rather quick adsorption process. Yu et al. [245] investigated the adsorption behavior of copper ions on graphene oxide chitosan (GO-CS) aerogels and found that adsorbent separation may be easily performed after adsorption using filtration or low-speed centrifugation. Furthermore, their findings revealed that GO–CS is an effective Cu^2+^ adsorbent, with a substantial adsorption capacity (254 mg/g) and a low binding strength.

### 6.9. Commercially Available Adsorbents

#### 6.9.1. Graphene

Due to the large surface area, improved active sites and functional groups nanomaterials are effective adsorbents for removing heavy metals from wastewater. Graphene is a two-dimensional carbon-based nanomaterial with a large specific surface area and excellent chemical stability. Due to its relatively large specific area, rich functional groups, and outstanding mechanical strength, graphene oxide (GO), one of the most notable graphene derivatives, has been shown to be a promising material for the preconcentration of heavy metal ions [246] GO, in particular, has a significant advantage in removing heavy metal ions from aqueous solutions, including lead (Pb (II)) [247], copper (Cu (II)) [248], cobalt (Co (II)) [249], cadmium (Cd (II)) [250], chromium [251]. Zhang et al. used chitosan–gelatin and GO to create ordered porous composites for Cu (II) and Pb (II) adsorption, and the composite had a very high ability to adsorb both metal ions [252]. Adsorption of high amounts of Pb (II) and Cd (II) onto GO was attributed to the GO’s higher surface area and oxygen functions [253]. Whereas, the adsorption of Cu (II) on GO was studied by Wu et al. and fit the experimental results to a Freundlich model with a maximum adsorption capacity of 117.5 mg/g for Cu (II). The adsorption of Cu (II) on GO was attributed to complexation, ion exchange, and electrostatic attraction [254].

Leng et al. investigated the use of graphene as an adsorbent for the removal of Sb (III) in aqueous solutions. Batch adsorption studies were carried out to investigate the impacts of operating conditions on Sb (III) adsorption, such as starting concentration, pH, and temperature. The Freundlich isotherm model appears to be followed rather than the Langmuir isotherm model. The adsorption capacity of graphene for Sb (III) was determined to be 10.92 mg/g under optimal circumstances [255]. Ren et al. investigated the Cu (II) and Pb (II) adsorption mechanisms on a graphene-MnO_2_ nanosheet. Adsorption data fit a Langmuir isotherm model well, indicating monolayer adsorption. With a 3 h equilibration time, the optimal adsorption capacities for Cu (II) and Pb (II) ions were found to be 1620 and 781 mol/g, respectively. The data were found to follow a pseudo-second-order kinetic model, indicating a chemisorption process [256]. Hao et al. used SiO_2_–graphene to study the adsorption of Pb (II) ions. The greatest percentage removal was 98.82% at pH 6 with a contact period of 30 min, and the optimal adsorption capacity was found to be 113.6 mg/g at 25 °C. The kinetics of adsorption followed a pseudo-second-order model. The Langmuir isotherm model fit the adsorption equilibrium data nicely [257]. Wu et al. investigated the ability of GO to remove Cu (II) ions from aqueous solutions by adsorption. Cu adsorption capability was found to be 117.5 mg/g in the GO (II) [258].

#### 6.9.2. Carbon Nanotubes

**Figure 12:**
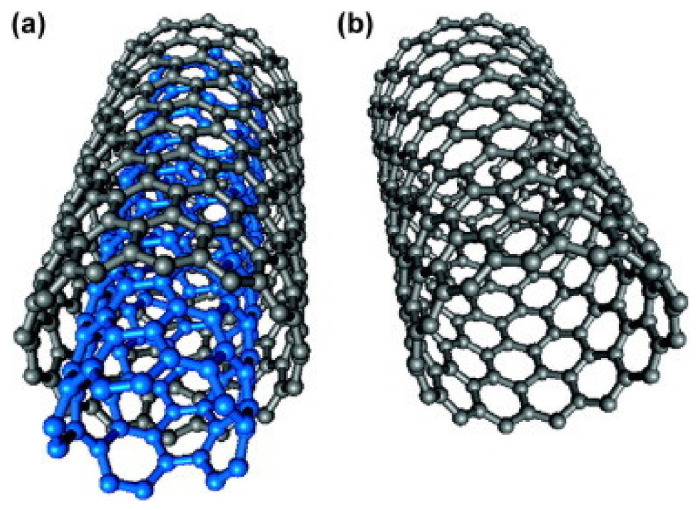
The structure of (**a**) MWCNT and (**b**) SWCNT [260].

Carbon has the ability to exist in a variety of molecular forms, which are referred to as allotropes of carbon. These allotropes can be thought of as various carbon structural variations. Carbon nanotubes (CNTs) are made up of cylindrical graphite sheets (an allotropic type of carbon) that have been rolled into a tube-like structure. Carbon nanotubes (CNTs), which belong to the fullerene structural family, are being studied for their ability to remove heavy metals from water (lead, chromium, cadmium, arsenic, copper, zinc, and nickel) [259]. Single-walled carbon nanotubes (SWCNTs) are cylindrical carbon nanotubes with a single graphene shell. Multi-walled carbon nanotubes (MWCNTs), on the other hand, are made up of many layers of graphene sheets. The structure of (a) multiwall carbon nanotubes (MWCNTs) and (b) single-walled carbon nanotubes is shown in Figure 12.

**Figure 13:**
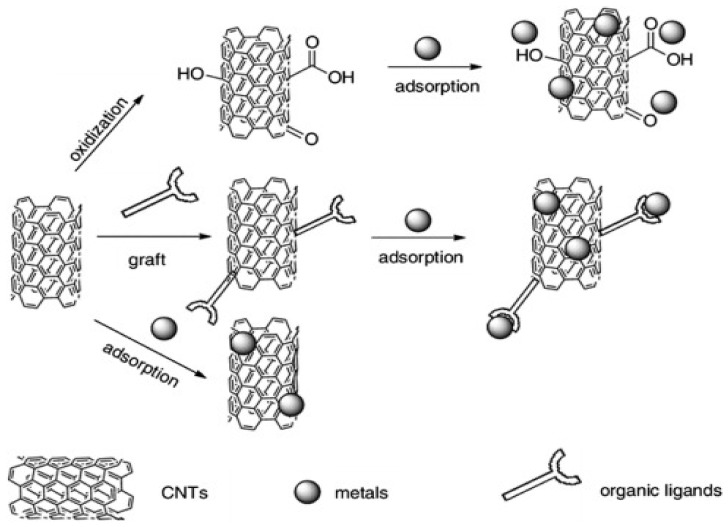
Schematic representation of CNTs grafting and metals adsorption [268].

Luo et al. (2013) found that MnO_2_ functionalised MWCNTs have a 41.6 mg/g adsorption capability for cadmium removal [261]. Liang et al. (2015) studied the adsorptive removal of cadmium on alumina-decorated MWCNTs and discovered a 27.21 mg/g adsorption capacity [262]. Furthermore, practically all studies show that the acid modified CNTs have a higher adoption capacity than the raw CNTs. This could be owing to an electrostatic interaction between the divalent heavy metal ions and the negative charge on the CNTs surface following acid treatment. CNTs are sometimes used in combination with other nanoparticles to boost the effectiveness of removal. Heavy metal ions have been successfully removed by modified carbon nanotubes [263,264]. Another technique to improve the surface features of CNTs is to graft functional groups or molecules onto them. Several studies have shown that CNTs grafted with various functional groups may remove heavy metals from aqueous solutions [265,266,267]. Figure 13 depicts a schematic of typical CNT surface grafting and subsequent metal ion adsorption.

**Figure 14:**
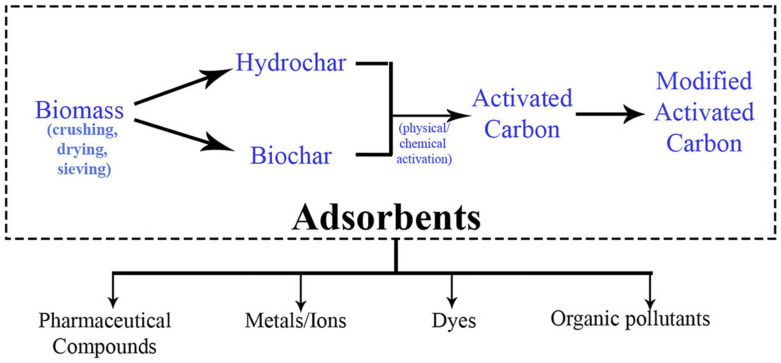
Synthesis routes of activated carbons from bio-waste.

In some research, the combined effect of electrostatic attraction and sorption–precipitation was shown to absorb metal ions [269]. Figure 14 showed possible methods for interacting cadmium ion adsorption on the surface of CNT/Al_2_O_3_. Cadmium ions are adsorbed on the surface of modified CNTs via physical interactions with the Cd (II) and hexagonally arranged carbon atoms in the graphite sheet. Cadmium ions are adsorbed in the graphite sheet. Yan et al. [270] study’s yielded promising results, demonstrating that MWCNTs treated with nitric acid may effectively remove metal ions from multicomponent systems. Maximal adsorption capacities, calculated by applying the equation of Langmuir for adsorption isotherms for the individual ions were 97.08 mg/g for Pb^2+^, 24.49 mg/g for Cu^2+^, and 10.86 mg/g for Cd^2+^.

#### 6.9.3. Activated Carbon

Activated carbon (AC) has been recognised as an essential sorbent for a long time. These adsorbents were synthesised by heat treatment of carbon substances followed by steam or chemical methods that drastically enhance the specific surface area and permeable character of the adsorbent [271]. Several adsorbents of activated carbons of various permeable sizes were obtained using various methods and were used for various applications in the extraction process. Coarse-activated carbon has also been used for the expulsion of harmful metals from groundwater. Walnut shell-based granular enacted carbon has additionally been discovered for evacuation and adsorption of color dissolved organic, heavy metals from wastewater [272]. ACs were synthesised by pyrolyzing polymer networks at high temperature followed by their physical or chemical activation (Figure 14).

Many polymers such as polyacrylonitrile, poly(styrene–DVB) sulphonate, polyvinyl chloride, polyvinylidene chloride, and phenolic resins have been utilised as starting materials for the formation of ACs [272,273]. These resins were potential adsorbents for harmful gases, fluids, and chemical warfare agents because of their permeable nature and high surface area. A unique adsorbent was synthesised by functionalising activated carbon with manganese oxide and tin oxide nanoparticles using formaldehyde as a crosslinker to bind them [274]. The maximum extraction efficiency of Pb^2+^, Cd^2+^ and Cu^2+^ ions on SnO_2_-NPs-AC-MnO2-NPs adsorbent were 2600, 2500 and 4100, μ mol/g at an initial concentration of Pb^2+^, Cd^2+^, and Cu^2+^ ions were 0.2 mol/L, time of sorption 30 min at pH 7 of the reaction medium and amount of adsorbent 5.0 mg of SnO_2_-NPs-AC-MnO_2_-NPs used. The batch method was used to find the extraction effectiveness Unwanted plants have been used to synthesise multi-pore activated carbons through the easy gradient heating method having a large area of the resultant sorbent [275]. The obtained sorbent has a high surface area containing multiple functionalities; this sorbent executes excellent adsorption properties for toxic heavy metal ions. The adsorbent was used to extract lead, cadmium, copper, nickel, and zinc metal ions from wastewater and sorption equilibrium was obtained within 150 min. Eeshwarasinghe et al. studied the removal of polycyclic aromatic hydrocarbons such as acenaphthylene, phenanthrene, and heavy metals like cadmium, copper, zinc by granular activated carbon [276]. Polycyclic aromatic hydrocarbons PAHs (initial concentration 1.0 ppm) showed better removal efficiency in comparison to toxic metal ions (initial concentration 20 ppm) at pH 5 with a sorbent quantity of 0.3 g per liter. The Langmuir adsorption value for both the PAHs was reduced with an enhanced concentration of metals, and the adsorption was found in the following pattern copper > zinc > cadmium. The activated sewage sludge was used as an adsorbent for dissolved pollutants. Three different kinds of sludge-based adsorbent were synthesised from the dried sludge: biochar/carbonised sludge (CS), sewage sludge-based activated carbon SBAC, and three modified MSBACs (MSBAC0, MSBAC4, and MSBAC10). They investigated the capacity of toxic ions to be extracted from carbonised sewage sludges produced by pyrolysis at 500 °C. The equilibrium adsorption values for sewage sludge-based activated carbon, MSBAC0, MSBAC4, and MSBAC10 obtained after 24 h were 1.25 mg/g, 3.04 mg/g, 3.4 mg/g, and 4.03 mg/g, respectively. Adsorption values of roughly 0.74 mg/g, 83% for SBAC adsorbent, were obtained after 5 min. With a sorption value of 1.1 mg/g, MSBAC0 had reached equilibrium after 1 min. The sorbents MSBAC4 and MSBAC10 demonstrated a higher sorption value for Pb (II) around 85% and 94.7% within 1 min [277]. Activated carbon derivative obtained through coconut buttons was used as a sorbent for extraction of lead, mercury, and copper from industrial waste material [278]. The batch method was used to study the adsorption capacity of the resultant adsorbent. It was found that adsorption value increased progressively with an increase in pH and the highest absorption was observed at pH 6.0 for lead and copper and 7.0 for mercury in the pH range 2.0–9.0. Experiments were conducted at a different initial concentration range from 25 to 100 ppm using 2.0 g/L activated carbon. It was found that removal efficiency increased with time and equilibrium was achieved after 3 h with a constant value of adsorption. Regeneration of adsorbent was done through 0.1 mol/L HCl and the adsorption-desorption cycle could be used three times for extraction. The removal ability of the resulting activated carbon followed this pattern: Pb^2+^, Hg^2+^, and Cu^2+^. Activated carbon from pecan nutshell was used for the elimination of Zn (II), Cd (II), Ni (II), and Cu (II), from a binary system of metal ions [279]. Dong et al., examined exhausted activated carbon for the extraction of heavy metal ions; the exhausted activated carbon was collected from a biological activated carbon water treatment plant of southern China [280]. It was found that activated carbon had a great sorption value of 95% and 86% for Pb^2+^ and Cd^2+^ at very little concentration of 200 ppb, respectively. Phosphoric acid impregnated activated carbon (DPAC) was synthesised from the seeds of date pits via single-step activation. Phosphoric acid is introduced into the seeds of date pits through 12-hour soaking [281]. After filtration, seeds were carbonised in a muffle furnace under continuous nitrogen flow. This process was done at 650 °C for 120 min. DPAC was utilised to remove lead ions with the maximum sorption capacity, which was around 101.35 mg/g at pH 6, in 30 min at 30 °C. The adsorbent was regenerated with hydrochloric acid and can be reused four times with a 10% reduction in lead ion extraction effectiveness. Iron oxide and iron oxide-activated carbon were prepared by the co-precipitation process for the extraction of Cr^6+^, Cu^2+^, and Cd^2+^ by using the batch method [282]. Different parameters affect the sorption rate such as pH (2.0,6.0), temperature (25 ± 1 °C), stirring rate 180 rpm, a dose of adsorbent (50 mg/10 mL), initial metal ions concentration (50 mg/L), and time for adsorption (3 h) was studied. Iron oxide nanoparticles were reported to achieve adsorption equilibrium for Cr^6+^, Cu^2+^, and Cd^2+^ after 60, 120, and 30 min, respectively. The extraction value of Cr^6+^ by activated carbon–iron oxide nanoparticles was obtained as 95.5% within 45 min after which no change in adsorption value occurs. Likewise, Cu^2+^ and Cd^2+^ removal efficiency was reached at 85.5%, 96.5% by the iron oxide/activated carbon nanoparticles within time periods of 90 min and 120 min.

## 7. Conclusions and Future Outlook

The potential of various adsorbents for the removal of heavy metals is demonstrated in this review. Adsorption is an economical method which has certain advantages over conventional methods such as being inexpensive, readily available, producing no sludge, being regenerated, and having technological feasibility, engineering applicability and affinity for the removal of heavy metals. Among the natural adsorbents like lignin, natural bentonite, tailored bentonic, the lignin was found to be the best adsorbent for lead ions (1865 mg/g), and chitosan was found to be a better adsorbent for cadmium ions with a removal capacity of 420 mg/g. It was also found that synthetic polymer poly(2-hydroxy ethyl methacrylate-methacrylolmidophenylalanine) showed good adsorption capacity for toxic metal ions like mercury (Hg = 669.4 mg/g), lead (Pb = 584.4 mg/g), cadmium (Cd = 268.4 mg/g), in comparison to poly(ethylene glycol dimetharylate-n-vinyl imidazole, poly(hydroxyethyl methacrylate-L-glutamic acid), acrylonitrile onto polypropylene fiber having a diethylene tri-amine group. Also, the isotherm and kinetics study reveals that the rate of sorption methods depends on the physical or chemical properties of the sorbent and is also influenced by various parameters like pH, time, amount of adsorbent, etc. The pseudo-second-order kinetic model best fit the majority of the processes, which follow the Langmuir or Freundlich isotherms. Furthermore, regeneration tests revealed that acidic eluents typically yield the highest desorption percentages. Usually, the rate of metal ions’ removal increased with time until the equilibrium was obtained for metal ions in the solid and liquid phases. From the current literature review, it is found that the sorption method is the best substitute for the traditional technology in extracting toxic metals. Additionally, more active functional groups on the adsorbent are required to remove a wide range of contaminants, maintain high mobility for an extended period of time, and meet the requirements for practical wastewater treatment. To change the adsorbent surface and boost toxin removal efficiency against a wide range of contaminants, certain dynamic compounds with high proclivity have been used. This review article offers a wide perspective on the use of different adsorbents but that are only applied at a laboratory scale. Further research is important to validate the finding on real industrial wastewaters for single and multi-pollutants.

## Figures and Tables

**Table 1:** Equilibrium modelling equations.

Langmuir Isotherm	Plot
Non-linear form	$Q_{e}$ = $\frac{Q_{m}\;K_{L}C_{e}}{1+K_{L}C_{e}}$
Type-I	$\frac{C_{e}}{Q_{e}}=\left(\frac{1}{Q_{m}}\right)\;C_{e}$ + $\left(\frac{1}{b_{L}Q_{m}}\right)$ $\frac{C_{e}}{Q_{e}}\;Vs\;C_{e}$
Type-II	$\frac{1}{Qe}=\left(\frac{1}{b_{L\;Q_{m}}}\right)\;\frac{1}{C_{e}}$ + $\frac{1}{Q_{m}}$ $\frac{1}{Q_{e}}\;Vs\;\frac{1}{C_{e}}$
Type-III	$Q_{e}=Q_{m}-\left(\frac{1}{b_{L}}\right)\;\frac{Q_{e}}{C_{e}}$ $Q_{e}\;Vs\;\frac{Q_{e}}{C_{e}}$
**Freundlich Isotherm**	
Non-linear form	$Q_{e}$ = $K_{F}$ $C_{e}\;$^1/n^
Linear form	ln ($Q_{e})$ = ln ($K_{F}$) + $\left(\frac{1}{n}\right)$ ln ($C_{e}$) ln ($Q_{e})\;Vs\ln\left(C_{e}\right)$
**Adsorption kinetics**	
(i) Pseudo-first order	dqt/dt = K_1_ (q_e_ − q_t_)
(ii) Pseudo-second order	dqt/dt = K (q_e_ − q_t_) ^2^
**Thermodynamics**	
	K_C_ = C_A_/Ce
	ΔG° = ΔH° − TΔS°
	ΔG° = −RTlinKc

Qm (mg/g) is the saturated monolayer adsorption capacity; bL (L/mg) is the constant related to the energy of sorption; Ce (mg/L) and Qe (mg/g) are the equilibrium liquid phase concentrations and amount of solute adsorbed at equilibrium, respectively; K_F_ is the constant related to adsorption capacity; n is the constant related to the adsorption intensity or degree of favourability of adsorption. qe and qt are the sorption capacity at equilibrium and at time t, K_1_ and K is the rate constant of pseudo-first order and -second-order constants. Kc is the constant of equilibrium, CA is the concentration of solid phase, Ce is the equilibrium concentration. ΔG° is the Gibbs free energy, ΔH° is the enthalpy change, ΔS° is the entropy change. T(K) is the absolute temperature, R is the gas constant (8.314 J/mol K).

**Table 2:** Comparison of adsorption capacities of natural adsorbents for removal of toxic metal ions.

S. No.	Adsorbent	Metal Ion/Adsorption Capacity	Reference
1	Lignin	Pb^2+^ = 1865 mg/g, Zn^2+^ = 95 mg/g	[107]
2	Chitosan (powder)	Cd^2+^ = 420 mg/g	[108]
3	Chitosan (beads)	Cd^2+^ = 518 mg/g	[108]
4	Seaweed brown algae	Cd^2+^ = 67 mg/g	[109]
5	*A. nodosum* seaweed	Cd^2+^ = 215 mg/g	[109]
6	Formaldehyde cross-linked *A. nodosum* seaweed	Cd^2+^ = 149 mg/g	[110]
7	Starch xanthate	Cd^2+^ = 33.3 mg/g Cr^2+^ = 17.6 mg/g Hg^2+^ = 1.15 mg/g	[111,112]
8	Cellulose xanthate	Cd^2+^ = 19.9 mg/g Cr^2+^ = 19.7 mg/g Hg^2+^ = 0.64 mg/g	[111]
9	Xanthated sawdust	Cd^2+^ = 21.4 mg/g Hg^2+^ = 30.1 ± 40.1	[112]
10	Zeolites	Pb^2+^ = 155.4 mg/g Cd^2+^ = 84.3 mg/g Cr^3+^ = 26.0 mg/g Hg^2+^ = 150.4 mg/g	[113]

**Table 3:** Different Polymeric adsorbents used for removal of various toxic metal ions.

S. No.	Adsorbent	Adsorption Capacity	Reference
1	Poly (ethylene glycol dimetharylate-n-vinyl imidazole)	1. Hg = 74.2 mg/g 2. Pb = 92.5 mg/g 3. Cd = 45.6 mg/g	[26]
2	Poly (vinyl pyrrolidinone)	1. Hg = 26.5 mg/g 2. Pb = 20.2 mg/g 3. Cd = 15.3 mg/g	[164]
3	Poly (hydroxy ethyl methacrylate-L-glutamic acid)	1. Hg = 26.8 mg/g 2. Pb = 42.5 mg/g 3. Cd = 17.6 mg/g	[165]
4	Poly (2-hydroxy ethyl methacrylate-methacrylolmidophenylalanine)	1. Hg = 669.4 mg/g 2. Pb = 584.4 mg/g 3. Cd = 268.4 mg/g	[165]
5	Acrylonitrile onto polypropylene fiber having diethylene triamine group	Hg = 657.9 mg/g	[166]
6	Polyurethane	1.Pb = 236.5 mg/g 2.Ni = 217.5 mg/g	[167]
7	Porous attapulgite (ATP)/polyethersulfone beads	1.Cu = 25.3 mg/g 2.Cd = 32.7 mg/g	[168]
8	amidoxime improved polyacrylonitrile (PAN) nanofibers	1.Cu = 52.70 mg/g 2. Pb = 263.45 mg/g	[169]

**Table 4:** Adsorption capacities of functionalised glycidyl methacrylate for removal of toxic metal ions.

S. No.	Adsorbent	Metal Ion	Adsorption Capacity	References
1	Amine-mercaptan modified poly (GMA-DVB)	Hg(II)	621.8 mg/g	[29]
2	(GMA-MMA-DVB) having pendant urea group	Hg(II)	7.8 mmol/g	[205]
3	Dibutylamine and chloroacetamide modified poly (GMA-EGDMA)	Hg(II)	2.5 mmol/g	[206]
4	Cross-linked Poly (GMA-aspartic acid)	Cu(II)	1.40 mmol/g	[207]
		Cd(II)	1.28 mmol/g	
5	Ethylenediamine modified poly(GMA--co-EGDMA)	Pt(IV)	1.3 mmol/g	[208]
6	Ferric oxide containing poly (GMA-MMA-EGDMA)	Hg(II)	124.8 mg/g	[29]
7	PGMA–EDA PGMA–DETA PGMA–TEPA	Hg (II)	4.76 mmol/g 4.80 mmol/g 4.21 mmol/g	[209]
8	PGMA–EDA PGMA–DETA PGMA–TEPA	Pb(II)	4.74 mmol/g 4.76 mmol/g 4.73 mmol/g	[209]

## Data Availability

Not applicable.

## Abbreviations

EIMH	(E)-2-[(1H-Imidazol-4-yl)methylidene]-Hydrazinecarbothioamide ligand
EDTA	Ethylene diaminetetra acetic acid
PADTC	Poly-ammonium dithiocarbamate
PSDTC	Poly-sodium dithiocarbamate
AC	Activated carbon
ATRP	Atom transfer radical polymerisation
SEM	Scanning electron microscopy
EDX	Energy dispersive X-ray
XRD	X-ray diffraction
SPGMA	Sulfonated poly(glycidyl methacrylate)
P(GMA-EGDMA)	Poly(glycidyl-methacrylate-ethylene glycol dimethylacrylate)
P(AMO-VPY)	Poly(4-acrylolmorpholine-co-4-vinyl pyridine)
P(DAPA-VPY)	Poly(3-dimethylamino)propylacrylate-co-4-vinyl pyridine
P(NDPPA-VPY)	Poly(N-3-dimethylamino)propyl methacrylamide-co-4-vinyl pyridine
